# Annelid phylogeny and the status of Sipuncula and Echiura

**DOI:** 10.1186/1471-2148-7-57

**Published:** 2007-04-05

**Authors:** Torsten H Struck, Nancy Schult, Tiffany Kusen, Emily Hickman, Christoph Bleidorn, Damhnait McHugh, Kenneth M Halanych

**Affiliations:** 1 Auburn University; Life Sciences Department; 101 Rouse Building; Auburn, AL 36849; USA; 2 University of Osnabrück; FB05 Biology/Chemistry; AG Zoology; Barbarastr. 11; 49069 Osnabrück; Germany; 3 Colgate University; Department of Biology; 204 Olin Hall; Hamilton, NY 13346; USA; 4 University of Potsdam; Institute of Biochemistry and Biology; Evolutionary Biology/Systematic Zoology; Karl-Liebknecht-Str. 24-25, 14476 Golm; Germany

## Abstract

**Background:**

Annelida comprises an ancient and ecologically important animal phylum with over 16,500 described species and members are the dominant macrofauna of the deep sea. Traditionally, two major groups are distinguished: Clitellata (including earthworms, leeches) and "Polychaeta" (mostly marine worms). Recent analyses of molecular data suggest that Annelida may include other taxa once considered separate phyla (i.e., Echiura, and Sipuncula) and that Clitellata are derived annelids, thus rendering "Polychaeta" paraphyletic; however, this contradicts classification schemes of annelids developed from recent analyses of morphological characters. Given that deep-level evolutionary relationships of Annelida are poorly understood, we have analyzed comprehensive datasets based on nuclear and mitochondrial genes, and have applied rigorous testing of alternative hypotheses so that we can move towards the robust reconstruction of annelid history needed to interpret animal body plan evolution.

**Results:**

Sipuncula, Echiura, Siboglinidae, and Clitellata are all nested within polychaete annelids according to phylogenetic analyses of three nuclear genes (*18S rRNA*, *28S rRNA*, *EF1α*; 4552 nucleotide positions analyzed) for 81 taxa, and 11 nuclear and mitochondrial genes for 10 taxa (additional: *12S rRNA, 16S rRNA*, *ATP8*, *COX1-3*, *CYTB*, *NAD6*; 11,454 nucleotide positions analyzed). For the first time, these findings are substantiated using approximately unbiased tests and non-scaled bootstrap probability tests that compare alternative hypotheses. For echiurans, the polychaete group Capitellidae is corroborated as the sister taxon; while the exact placement of Sipuncula within Annelida is still uncertain, our analyses suggest an affiliation with terebellimorphs. Siboglinids are in a clade with other sabellimorphs, and clitellates fall within a polychaete clade with aeolosomatids as their possible sister group. None of our analyses support the major polychaete clades reflected in the current classification scheme of annelids, and hypothesis testing significantly rejects monophyly of Scolecida, Palpata, Canalipalpata, and Aciculata.

**Conclusion:**

Using multiple genes and explicit hypothesis testing, we show that Echiura, Siboglinidae, and Clitellata are derived annelids with polychaete sister taxa, and that Sipuncula should be included within annelids. The traditional composition of Annelida greatly underestimates the morphological diversity of this group, and inclusion of Sipuncula and Echiura implies that patterns of segmentation within annelids have been evolutionarily labile. Relationships within Annelida based on our analyses of multiple genes challenge the current classification scheme, and some alternative hypotheses are provided.

## Background

Annelids are found throughout the world's terrestrial, aquatic, and marine habitats, and are the most abundant component of the macrofauna in the deep sea. As one of three major animal groups with segmentation, annelids are critical in any investigation of body plan evolution; we need to understand the composition and branching history of groups within the annelid radiation if we are to progress towards elucidation of the last common ancestor of bilaterians and evolution of segmentation. Surprisingly, annelid evolution is poorly understood. To rectify this situation, data sets of multiple genes are being used to evaluate diversity and relationships of major annelid clades.

Annelida is part of the lophotrochozoan radiation that includes Mollusca, Brachiopoda, Bryozoa, Nemertea and Sipuncula [1]. Traditionally classified as "Polychaeta" and Clitellata, three taxa historically assigned phylum status have been hypothesized to also fall within Annelida: Sipuncula, Echiura, and Siboglinidae (previously known as Pogonophora and Vestimentifera). Sipunculan origins are the most controversial of these three; some morphological data suggest molluscan affiliations [2], but a growing body of data indicates annelid affinities for this group of unsegmented marine worms [3-7].

Both molecular and morphological data support Echiura and Siboglinidae as annelids [7-14]. Echiura possess annelid-like features such as the ultrastructure of cuticle and chaetae, the development of the mesoderm, and structure and position of the blood vessels [15]; additionally, their larval nervous system indicates a possible segmented ancestry even though overt segmentation is lacking in adults [13]. Recent *18S rRNA *analyses support a sister relationship of Echiura and capitellid polychaetes [12,16,17], while an analysis of five gene regions (*18S*, D1 & D9-10 of *28S*, *histone H3*, *snU2 RNA*, *COX1*) by Colgan et al. [7] supports a sister group relationship with Terebelliformia, indicating that the former may reflect a single gene artifact. Recent analyses of four gene regions (*18S rRNA*, D1 of *28S RNA*, *histone H3*, *16S rRNA*) are inconclusive; one analysis favors a sistergroup relationship to Capitellidae and to *Protodrilus purpureus *and Pectinaridae in the other one [18].

The ultrastructure of the uncini (short, apically toothed chaetae) implies a closer relationship of Siboglinidae with the annelid taxa Terebelliformia and Sabellida [10], and Rouse and Fauchald [11] (R&F) presented a cladistic analysis of morphological characters that corroborates this. The placement of siboglinids with annelids is also supported, albeit weakly, by several molecular studies (mostly based on *18S rRNA*) [e.g., [5-8,12,19-22]]. None of these hypotheses has been explicitly tested using a rigorous statistical framework.

Analyses of morphological and molecular data support clitellate monophyly and have provided robust hypotheses of relationships within this taxon [23-25]. However, monophyly of "Polychaeta" lacks support and resolving polychaete annelid relationships has been difficult [26,27]. Although some authors regard "Polychaeta" as sister to Clitellata [e.g., [11]], increasing evidence based on molecular and morphological data suggests they are a paraphyletic grade including clitellates [e.g., [3,8,12,20,23,28-30]].

Polychaete annelids have been classified into approximately 80 families, generally supported as monophyletic, but arrangement of these into well-supported more-inclusive nodes is wanting [31]. R&F's [11] analyses provide the most taxonomically inclusive hypotheses of polychaete relationships to date. Based on morphological cladistic analyses of polychaete families, they proposed a monophyletic "Polychaeta" consisting of two major clades, Scolecida and Palpata, the latter divided into Canalipalpata and Aciculata. While these analyses provide objective and testable hypotheses of polychaete relationships, and thus significantly moved the field forward, many aspects of our current understanding of annelid phylogeny are still poorly understood. Monophyly of Scolecida, Palpata and Canalipalpata has been questioned by some morphologists [30,32,33], and uncertainties of character scoring, homology assessment, and difficulty in rooting of the annelid tree make resolution of annelid relationships using morphology unlikely [27,30,34,35]. Molecular analyses to date show no evidence to support the classification scheme developed from the R&F analyses, but most molecular studies have been based on single genes [see [16,20,26,27]]. In a recent study that included the most comprehensive annelid taxon sampling to date, incomplete character data for the four genes analyzed may explain the lack of resolution [18]; of the 217 taxa, only 52 had data for all genes, and over 50 were represented by data for a single gene only. Neither molecular nor morphological studies have yet convincingly eliminated the possibility that these clades are monophyletic despite the doubts raised.

To address these major outstanding issues of annelid inclusiveness and phylogeny, we reconstruct relationships of major annelid and lophotrochozoan taxa using two data sets. One data set is built on ~6.5 kb of sequence from three nuclear genes [nuclear small and large ribosomal subunits (*18S rRNA *and *28S rRNA*), and elongation factor-1α (*EF1α*); referred to as the Nuc data set] for 81 taxa that span 45 traditional polychaete families as well as Siboglinidae, Echiura, Clitellata, and Sipuncula and nine outgroup OTUs to address both annelid inclusiveness and phylogeny. The other data set comprises the three nuclear genes and eight mitochondrial genes (~13.4 kb) for a restricted taxon sampling of 10 operational taxonomic units (OTUs) to address annelid inclusiveness with respect to Sipuncula, Echiura and Siboglinidae [referred to as the NucMt data set]. These are the two largest data sets for annelids explored to date in terms of amount of numbers of characters for a full range of taxa, and it is our hope that the taxonomic representation of these datasets will continue to grow in order to provide a more holistic comparison to morphological hypotheses. Besides analyses using Maximum likelihood (ML) and Bayesian inference (BI) *a priori *hypotheses about annelid relationships were explicitly tested against best trees using approximately unbiased (AU) and non-scaled bootstrap probability (NP) tests.

## Results and Discussion

### Annelida includes Echiura, Siboglinidae, and Sipuncula

**Figure 1:**
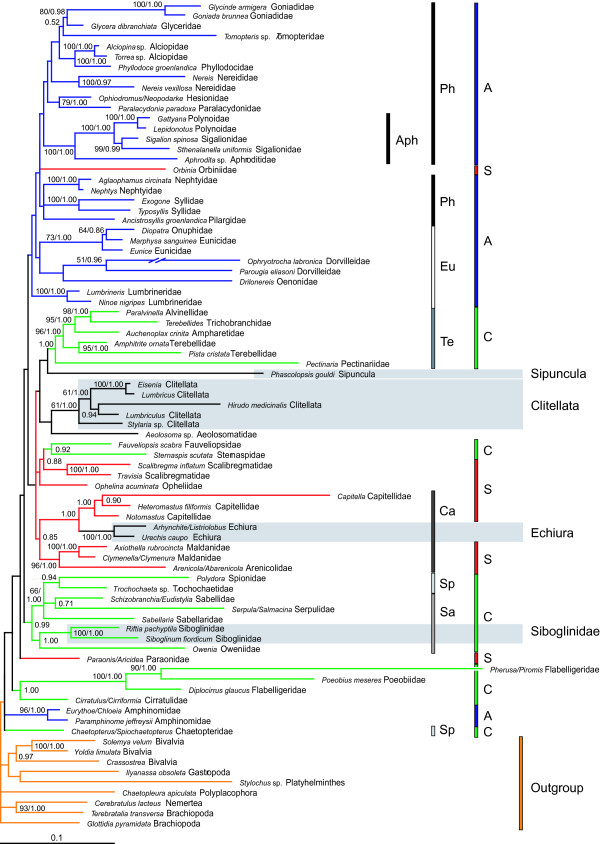
ML analysis and BI of Nuc data set with 81 OTUs (-ln L = 66,627.30). 1 of 2 best trees is shown. In the other tree the trichotomy of Nephtyidae, Syllidae, and Pilargidae is resolved with Syllidae being sister of Nephtyidae. OTUs with just the genus names (e.g., *Lumbricus*) indicate that the sequences from different species of that genus were concatenated. Nuc consisted of 9,482 characters, from which 4,552 (*28S rRNA *– 2,504; *18S rRNA *– 1,375; *EF1α *– 673) unambiguously aligned and non-saturated ones were included. BS values above 50 shown at the branches on the left; PP's on the right or alone. The branch leading to *Ophryotrocha labronica *is reduced by 90%. *Ophryotrocha *individuals have been sampled from a long time culture, which got bottlenecked several times. For *28S rRNA*, *Capitella *forms a long branch and does not cluster with the two other Capitellidae in the analyses. ML settings: Base frequencies: A = 0.2727, C = 0.2495, G = 0.2586, T = 0.2192; Rate matrix: AC, AT, CG, GT = 1.0000, AG = 2.5097, CT = 3.7263; α = 0.4830; Proportion of invariant sites = 0.3103. Models in BI: *28S rRNA*, *18S rRNA*, *EF1α *: GTR+I+Γ. Clitellata, Echiura, Siboglinidae, Sipuncula highlighted with gray and bars indicate polychaete groups: orange = outgroup; A, blue = Aciculata; C, green = Canalipalpata; S, red = Scolecida; Ca = Capitellida; Eu = Eunicida; Ph = Phyllodocida; Sa = Sabellida; Sp = Spionida; Te = Terebelliformia; Aph = Aphroditiformia.

**Figure 2:**
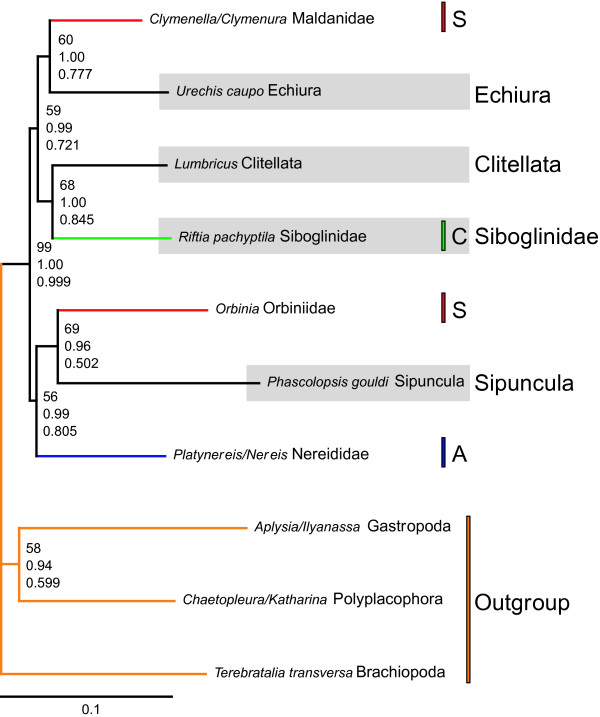
ML analysis and BI of NucMt data set with 10 OTUs and only nucleotides (-ln L = 29,951.42). OTUs with just the genus names (e.g., *Lumbricus*) indicate that the sequences from different species of that genus were concatenated. NucMt consisted of 16,351 characters with 11,454 unambiguously aligned and non-saturated (in addition to Nuc: *COX1 *– 489; *16S rRNA *– 463; *18S rRNA *– 406; *CYTB *– 365; *COX3 *– 258; *COX2 *– 219; *NAD6 *– 118; *ATP8 *– 32). BS values shown at upper position right to node; PP's in the middle; 1-*p *of AU test at lower position. ML settings: Base frequencies: A = 0.2536, C = 0.2243, G = 0.2498, T = 0.2723; Rate matrix: AC = 2.3662, AG = 4.2648; AT = 2.0966, CG = 2.5643, CT = 6.7166, GT = 1.0000; α = 0.4824; Proportion of invariant sites = 0.3555. Models in BI: *28S rRNA*, *12S rRNA*: GTR+I+Γ ; *18S rRNA*: K80+I+Γ ; *EF1α *= F81+I+Γ ; *16S rRNA*, *CYTB*: GTR+Γ ; *ATP8*, *COX1-3*, *NAD6 *= F81+Γ. Clitellata, Echiura, Siboglinidae, Sipuncula highlighted with gray and bars indicate polychaete groups: orange = outgroup; A, blue = Aciculata; C, green = Canalipalpata; S, red = Scolecida.

Phylogenetic analyses of 153 new and 100 previously published sequences for individual genes and combined data sets (Figs. 1, 2, and Additional files 1, 2, 3, 4) all indicate Annelida is much more diverse than traditionally recognized, and includes Sipuncula, Echiura, and Siboglinidae, thus confirming previous suggestions. The Nuc and NucMt data sets place sipunculans, echiurans, and siboglinids within an otherwise monophyletic Annelida (Figs. 1, 2, Additional file 4), suggesting that body plan segmentation is evolutionarily labile. Although nodal support was weak deep in the Nuc tree (Fig. 1), this more inclusive annelid clade is significantly supported in all NucMt analyses (bootstrap value (BS) = 99; PP = 1.00 for both protein-coding genes coded as either nucleic or amino acids; 1-*p *value of the AU test = 0.999; Figs. 2 & Additional file 4). Furthermore, our conclusions are significantly supported when we explicitly tested *a priori *hypotheses against the best trees using AU and NP tests of both the Nuc and NucMt data sets (Table 1). Whereas the results of the *28S rRNA *analyses are consistent with those of the Nuc and NucMt data sets (PP = 0.85) [see Additional file 1], separate analyses of *18S rRNA *and, to a lesser degree, *EF1α *show unexpected placement of outgroups throughout Annelida (e.g., Mollusca) [see Additional files 2 &3]. None of these outgroup relationships are significantly supported; presumably phylogenetic signal in this region of the tree is limited when these genes are analyzed individually, a phenomenon well documented for *18S rRNA *[1]. Therefore, we focus on the Nuc and NucMt analyses.

**Table 1:** Results of the approximately unbiased (AU) and the non-scaled bootstrap probability (NP) tests of the Nuc and NucMt data sets.

	**Nuc**			**NucMt**		
	*Difference to best trees*	*au*	*np*	*Difference to best tree*	*au*	*np*
Best Tree(s)		0.691	0.322		0.771	0.586
		0.622	0.297			
Sipuncula excluded	12.7	0.517	0.228	3.2	0.483	0.361
Sipuncula & Mollusca	32.0	0.187	0.073	**23.0**	**0.010**	**0.004**
Echiura excluded	**183.3**	3*10^**-6**^	2*10^**-6**^	**17.7**	**0.047**	**0.022**
Echiura sister to Terebelliformia	**147.0**	3*10^**-46**^	6*10^**-16**^			
	**147.0**	3*10^**-46**^	6*10^**-16**^			
Siboglinidae excluded	48.7	0.060	**0.022**	**41.8**	**0.006**	**0.001**
Clitellata excluded	**106.7**	**0.003**	9*10^**-5**^	**20.2**	**0.039**	**0.014**
Scolecida (including Echiura)	30.0	0.175	0.055	**22.0**	**0.015**	**0.005**
Palpata	**129.7**	6*10^**-84**^	4*10^**-21**^			
Aciculata	**55.2**	**0.022**	**0.005**			
Canalipalpata	**79.4**	3*10^**-4**^	6*10^**-5**^			

Significant differences (*p *< 0.05) in bold.

The hypothesis of a Sipuncula/Mollusca relationship is rejected by AU and NP tests for NucMt and thus corroborates some previous reports [3,5,6,36]. These results agree with previous studies suggesting that the molluscan cross organization of micromeres during spiral cleavage should not be regarded as synapomorphic for mollusks and sipunculans [2,37]. Wanninger et al. [38] discussed the possession of a ventral median nerve as additional morphological support for a sipunculid-annelid relationship, but these nerves are also observed in other taxa like Gnathostomulida [39-41]. The position of Sipuncula within the annelid radiation varies among analyzed data sets (Figs. 1, 2, Additional files 1, 2, 3, 4) and we were not able to significantly discriminate via the AU or NP test whether sipunculans were sister to or derived within Annelida (Table 1). However, in all of the resulting 81-OTU trees (Figs. 1, Additional files 1, 2, 3) sipunculans were nested within annelids, and never placed as a sister taxon, consistent with the NucMt results and some previous studies [6,7]. Interestingly, the lining of nephridial podocytes is a morphological feature shared by Sipuncula and Terebelliformia that bolsters the close, albeit weak association between these two groups in the 81-taxon tree (Fig. 1) [42].

Echiuran inclusion within the annelids, first suggested by *EF1α *data [8], was significantly supported in our analyses (Figs. 1 &2, Table 1). Moreover, all of our analyses of individual genes or combined data with 81 OTUs, placed echiurans as sister to Capitellidae [*18S rRNA *(BS: 95; PP: 1.00), *28S rRNA *(BS: 100; PP: 1.00 both excluding *Capitella*), *EF1α *(BS < 50; PP: 0.56), and Nuc (BS = 99 excluding *Capitella*; PP = 1.00), note that *Capitella 28S rRNA *may have long branch issues] confirming earlier suggestions [12,16-18,20], and in sharp contrast to recent analyses of several gene regions placing echiurans with Terebelliformia taxa [7]. Indeed, our explicit hypothesis testing significantly rejects such a close relationship (Table 1). In the Nuc analyses, Echiura/Capitellidae is sister to a scolecidan clade of Maldanidae and Arenicolidae clade (Fig. 1); this result is echoed in the NucMt data, which lacks a capitellid but places the echiuran and maldanid as sister taxa (Fig. 2). Due to this placement of Echiura as a derived annelid group, interpretations of nerve development in echiurans as vestigial segmentation are fully warranted [13].

The long-held hypothesis that Siboglinidae (including vent worms, pogonophorans and the recently discovered bone eating worms *Osedax *[43]) are within Annelida [e.g., [8,10-12,14,21]] is supported with significant statistical rigor (Table 1). Our results corroborate previous molecular studies supporting a nested position of Siboglinidae within Annelida (Figs. 1, 2, Additional files 1, 2, 3, 4) [8,12,14]. Furthermore, the Nuc data set and *28S rRNA *partition (Figs. 1, Additional file 1) show a close, albeit weak relationship between Siboglinidae and Sabellida, specifically Oweniidae, as suggested by analyses of morphological data [10,11], some molecular data [44] and combined analyses of both [21]. The ultrastructure of the uncini and the possession of only one pair of nephridia with dorsal pores in the first segment are possible synapomorphic features [10]. Morphological characters supporting the sister group relationship of Oweniidae and Siboglinidae are neuropodial chaetae in posterior segments emerging straight from the body wall and an intraepidermal nerve cord [21]. The close relationship to Clitellata as shown by the NucMt data set (Fig. 2) is not substantiated by morphological data.

### Position of Clitellata

In all analyses, Clitellata is derived from polychaetous ancestors; it does not form a major basal branch of annelids (Figs. 1, 2, Additional files 1, 2, 3, 4). This placement is significantly supported by hypothesis testing (Table 1), and corroborates a series of previous studies [see for review [20,23,27]]. In all analyses with 81 OTUs except those of the *EF1α *partition, the small mainly freshwater meiofaunal aeolosomatid *Aeolosoma *sp. is sister to Clitellata; however, the support for this relationship is weak (Figs. 1, Additional files 1 &2). Previous authors have discussed *Aeolosoma *as either a highly derived clitellate [45,46], as sister to or the most basal clitellate (depending on which morphological characters are used to define Clitellata) [47-52], or as not closely related to Clitellata [20,28,53-55]. Our findings strengthen the conclusions of previous but more limited studies [47-52], and offer further evidence of a freshwater origin of Clitellata [24]. However, additional sampling of terrestrial and freshwater polychaetes would be needed before the relationship between *Aeolosoma *and Clitellata can be considered resolved.

### Polychaete Relationships

While the relationships of polychaetous annelids are still poorly understood, our testing of the four major polychaete clades proposed by Rouse and Fauchald [11] are all significantly rejected by our analyses (Table 1). These results provide rigorous support for previous *ad hoc *arguments based on morphological and molecular data [7,16,18,20,26,27,30,32,33]. Due to the strong affiliation of echiurans with capitellids, we opted to provisionally include echiurans within Scolecida during hypothesis testing to understand whether that group was natural regardless of echiuran position. Rejection of Scolecida, Palpata and Canalipalpata is not surprising due to dispersion of their taxa throughout the tree (Fig. 1) and given that support of these groups by morphological data is weak [30,32,33]. For example, the presence of palps is doubted in some Palpata group members (Ampharetidae, Pectinariidae and Terebellidae), and palp nerves but no protruding palps have been shown in some scolecids (Scalibregmatidae and Paraonidae) [56].

Aciculata was the least problematic of the R&F taxa, in that it was the exclusion of only two taxa that precluded its monophyly on the tree. For the Nuc data, rejection of Aciculata by AU and NP tests is due to the placement of *Orbinia *within Aciculata (orbiniids are considered scolecids) and the placement of amphinomids at the base of the tree (Fig. 1). These results are in contrast to two recent multi-gene analyses where the Aciculata taxa Eunicida and Phyllodocida are dispersed throughout the trees [7]. Interestingly, supportive chaetae of Orbiniidae may be homologous with the acicula of Aciculata [33], and Amphinomida has been regarded as basal based on the tetraneurous organization of the nervous system [57,58].

As in previous studies encompassing polychaete taxa [e.g., [7,12,20]], nodal support above the family level is weak (BS < 50; PP < 0.95). Nonetheless, some taxa recovered by R&F's [11] analyses are also supported in our analyses (Fig. 1). For example, the traditional Capitellida consisting of Capitellidae, Arenicolidae and Maldanidae is recovered, but includes Echiura; Terebelliformia and Aphroditiformia are also recovered; and a clade encompassing all Sabellida and Spionida except Chaetopteridae appears in the tree. In contrast to Rousset et al. [18] and Colgan et al. [7], monophyly of Annelida including Echiura and Sipuncula is also revealed by all combined analyses (Fig. 1, 2, Additional file 4). Thus, with an increasing number of characters our understanding of annelid phylogeny and inclusiveness is progressing, even using ribosomal rRNA genes. A similar effect has been observed for protostomes, lophotrochozoans and eunicidans as well [4,59,60].

### Evolution of body plans

Segmentation has traditionally been thought to be a conserved morphological character complex supporting an Arthropoda plus Annelida clade, "Articulata"; an implicit assumption has been that homoplasy of segmentation is unlikely. Molecular phylogenetic analyses favor Lophotrochozoa and Ecdysozoa and reject Articulata with steadily increasing support, which implies convergence of their body plans and specifically of the complex "segmentation" [see [1]]. Interestingly, recent studies of Annelida and Arthropoda central nervous systems reveal a higher variability than previously expected and a typical rope ladder-like central nervous system, a key feature of the complex "segmentation" cannot be found [see [56,61]]. Our results provide statistical support for trees that indicate a high plasticity in the evolution of this fundamental body plan character in annelids: the placement of unsegmented Echiura and Sipuncula within Annelida implies that these two groups have independently lost segmentation; and members of Siboglinidae possess highly modified segmentation that is only obvious in their posterior ophistosoma.

## Conclusion

The molecular phylogenetic analyses presented here corroborate previous studies in suggesting a very different view of annelid evolution than is traditionally accepted; the tests of alternative hypotheses provide statistical support for our conclusions. Sipuncula is most likely a derived annelid or the annelid sister group. Echiura is the sister group to Capitellidae as revealed by three nuclear markers and their exclusion from Annelida is significantly rejected. Accepting Annelida as including Echiura and Sipuncula not only suggests that segmentation is more evolutionary labile than previously assumed, but that other characters distinctive of annelids, such as complex chaetae comprised of β-chitin and arranged in four groups, have also been reduced or secondarily lost in some of these derived taxa [23].

Hypothesis testing clearly rejects the exclusion of Clitellata and Siboglinidae from "Polychaeta", and monophyly of Scolecida, Palpata, Canalipalpata or Aciculata. Furthermore, some higher level annelid taxa are suggested by our analyses, i.e., Aphroditiformia, Terebelliformia, Sabellida/Spionida excluding Chaetopteridae, Capitellida including Echiura, Eunicida/Phyllodocida including Orbiniidae. However, we need to progress further in the resolution of annelid phylogeny, and we recognize that data from additional genes and for more complete taxonomic representation of annelids may robustly resolve the basal nodes in the annelid tree and elucidate the precise positions of Sipuncula, Clitellata, and Siboglinidae.

## Methods

### Data assembly

Additional file 5 lists taxa and genes employed, and GenBank accession numbers. Upon collection, samples were preserved in > 70% non-denatured Ethanol, RNAlater (Invitrogen) or frozen at 80°C. Procurement of *18S rRNA *and *28S rRNA *sequence data followed Struck *et al*. [60]. For *EF1α*, total RNA was isolated using RNAwiz™ (Ambion) and reverse transcribed using SuperScript™ II (Invitrogen). Amplification of *EF1α *followed McHugh [8] with an additional primer (JH16R: 5'-KNRAANKNYTCNACRCACA-3') using touchdown PCR and a second round of amplification. Further amplification details can be found in Supplementary Data. The TOPO TA Cloning Kit for Sequencing (Invitrogen) was used to clone most *EF1α *products. An ABI Prism 310 Genetic Analyzer and Big Dye Terminator v.3.1 (Applied Biosystems) were used in sequencing.

### Phylogenetic Analyses

A data set (NucMt) consisting of eight mitochondrial genes (*COX1-3*, *CYTB*, *NAD6*, *ATP8*, *12S *and partial *16S*), as well as three nuclear genes *18S rRNA*, *28S rRNA *and *EF1α *were analyzed using the 7 available ingroup mitochondrial genomes plus 3 outgroups (2 mollusks and 1 brachiopod). Five mollusks, two brachiopods, a nemertean and a platyhelminth were outgroups for individual genes and the concatenated (Nuc) data sets. Sequences were aligned with CLUSTAL W [62] using default settings and corrected by hand. Ambiguous positions were excluded from the subsequent analysis. The alignments (Accession #S1766; Nuc matrix #M3221 and NucMt data set #M3220 & #M3219) are available at TREEBASE. To assess phylogenetic signal, regions within genes were analyzed using a procedure modified from Jördens *et al*. [17]. Saturated positions were removed. χ^2 ^tests did not reject homogeneity of base frequencies across taxa [see Additional file 6]. Appropriate ML models for each of the 5 data sets [see Additional file 7] were determined with Modeltest V 3.06 [63,64]. PAUP*4.0b [65] using heuristic searches, tree-bisection-reconnection (TBR) branch swapping, 10 random taxon additions and model parameters reconstructed topologies. Nodal support was estimated by 100 BS replicates with 10 random taxon addition and TBR branch swapping for the NucMt data set. However, for the 81-OTU data sets ML bootstrapping using heuristic searches is not applicable due to computational time burden. Therefore, each 81-OTU data set was analyzed using 10,000 BS replicates, neighbor-joining searches and ML settings for the parameter specification.

For BI, MrModeltest 1.1b [66] determined prior probability distributions of individual parameters of nucleotide substitution models for each gene partition in MrBayes 3.1 [67]. The mixed amino acid substitution model option was chosen for each protein-coding gene partition when analyzing amino acid sequences in the NucMt data. Partitions were unlinked. Analyses employed two runs with three heated and one cold chain started simultaneously for either 1*10^7 ^generations (*18S rRNA *and *28S rRNA*), 5*10^6 ^generations (*EF1α *and Nuc), or 1*10^6 ^generations (NucMt, protein-coding genes coded as either nucleotides or amino acids), with trees being sampled every 250 generations. Based on convergence of likelihood scores, *burnin *trees were discarded (*18S rRNA*: 6,760 trees; *28S rRNA*: 28,000; *EF1α *: 10,000; Nuc: 8,000; NucMt, both: 201) and posterior probabilities determined from remaining trees.

### Hypothesis Testing

Significance tests using both the AU and the NP test of CONSEL [68,69] were performed under the ML criterion for the Nuc and NucMt data sets for several *a priori *hypotheses against the best trees. The following hypotheses were tested for Nuc and NucMt data sets: 1) Sipuncula is not an annelid, 2) Sipuncula and Mollusca are closely related, 3) Echiura is not an annelid, 4) Siboglinidae is not an annelid, 5) Clitellata is not a subtaxon of polychaetes, 6) monophyly of Scolecida including Echiura; and additionally for the Nuc data set a sister group relationship of Echiura and Terebelliformia as well as monophyly of the other three major polychaete clades (Palpata, Aciculata, Canalipalpata). To obtain the best result for each *a priori *hypothesis the analyses were constrained by allowing only trees congruent with the particular *a priori *hypothesis. Due to strong support for a Capitellidae/Echiura sistergroup relationship with *18S rRNA *data sets, the taxon assemblage for Scolecida was changed from R&F [11] to include Echiura biasing the test in favor of supporting Scolecida. Furthermore, each clade obtained in NucMt nucleotide only analyses were compared against the best alternative hypothesis not congruent with the clade by means of AU tests resulting in the 1-*p *values of Fig. 2. This is an approach similar to decay indices in Parsimony analyses [70,71], but with actual significance values; *p *< 0.05 shows significant difference between the two alternative clades. Thus, in congruence with BS and PP values 1-*p *has to be greater than 0.95.

## List of Abbreviations

*ATP8 *– *ATP synthase subunit 8*

AU – approximately unbiased

BI – Bayesian inference

BS – bootstrap

*CYTB *– *cytochrome b*

*COX1 *– *cytochrome c oxidase subunit I*

*COX2 *– *cytochrome c oxidase subunit II*

*COX3 *– *cytochrome c oxidase subunit III*

*EF1α *– *elongation factor-1α*

ML – maximum likelihood

*NAD6 *– *NADH dehydrogenase subunit *6

NP – non-scaled bootstrap probability

Nuc – nuclear genes only

NucMt – nuclear and mitochondrial genes

OTU – operational taxonomic unit

PP – posterior probabilities

R&F – Rouse & Fauchald

TBR – tree-bisection-reconnection

## Authors' contributions

K. M. H. and D. M. H. conceived this study. T. H. S. took the lead on *18S rRNA *and *28S rRNA *data collection, analyses and writing. N.S. took the lead with EF1-α data collection and aided overall organization. C. B., T. K., E. H. collected data. T. H. S., N. S., C. B., D. M. H. and K. M. H. all contributed to writing the manuscript. All authors read and approved the final manuscript.

## Supplementary Material

Additional file 1 ML tree of *28S rRNA *partition. This file contains the result of the phylogenetic reconstruction of the *28S rRNA *partition with 81 OTUs. Click here for file

Additional file 2 ML tree of *18S rRNA *partition. This file contains the result of the phylogenetic reconstruction of the *18S rRNA *partition with 81 OTUs. Click here for file

Additional file 3 ML tree of *EF1α *partition. This file contains the result of the phylogenetic reconstruction of the *EF1α *partition with 81 OTUs. Click here for file

Additional file 4 BI tree of the NucMt data set with amino acid partitions. This file contains the result of the phylogenetic reconstruction of the NucMt data set with 10 OTUs and protein-coding genes translated into amino acid sequences using the genetic standard code for *EF1α *and the mitochondrial invertebrate code for the mitochondrial genes *ATP8*, *COX1-3*, *CYTB*, and *NAD6*. Click here for file

Additional file 5 List of taxa. This file contains a list of taxa used in the phylogenetic analyses. Click here for file

Additional file 6 Detection of saturated positions. This file contains further information on amplification of *EF1α*, alignment procedures, homogeneity of base frequencies and phylogenetic results of data partitions. Click here for file

Additional file 7 Substitution models used in analyses. This file contains a list of Models used in Maximum Likelihood and Bayesian Inference analyses. Click here for file
