# Supplementary material for: Annelid phylogeny and the status of Sipuncula and Echiura
Source: BMC Evol Biol. 2007 Apr 5;7:57. doi: 10.1186/1471-2148-7-57 (PMC1855331; doi:10.1186/1471-2148-7-57)
Supplement: Additional file 1 — ML tree of 28S rRNA partition. This file contains the result of the phylogenetic reconstruction of the 28S rRNA partition with 81 OTUs. [file 1471-2148-7-57-S1.pdf]

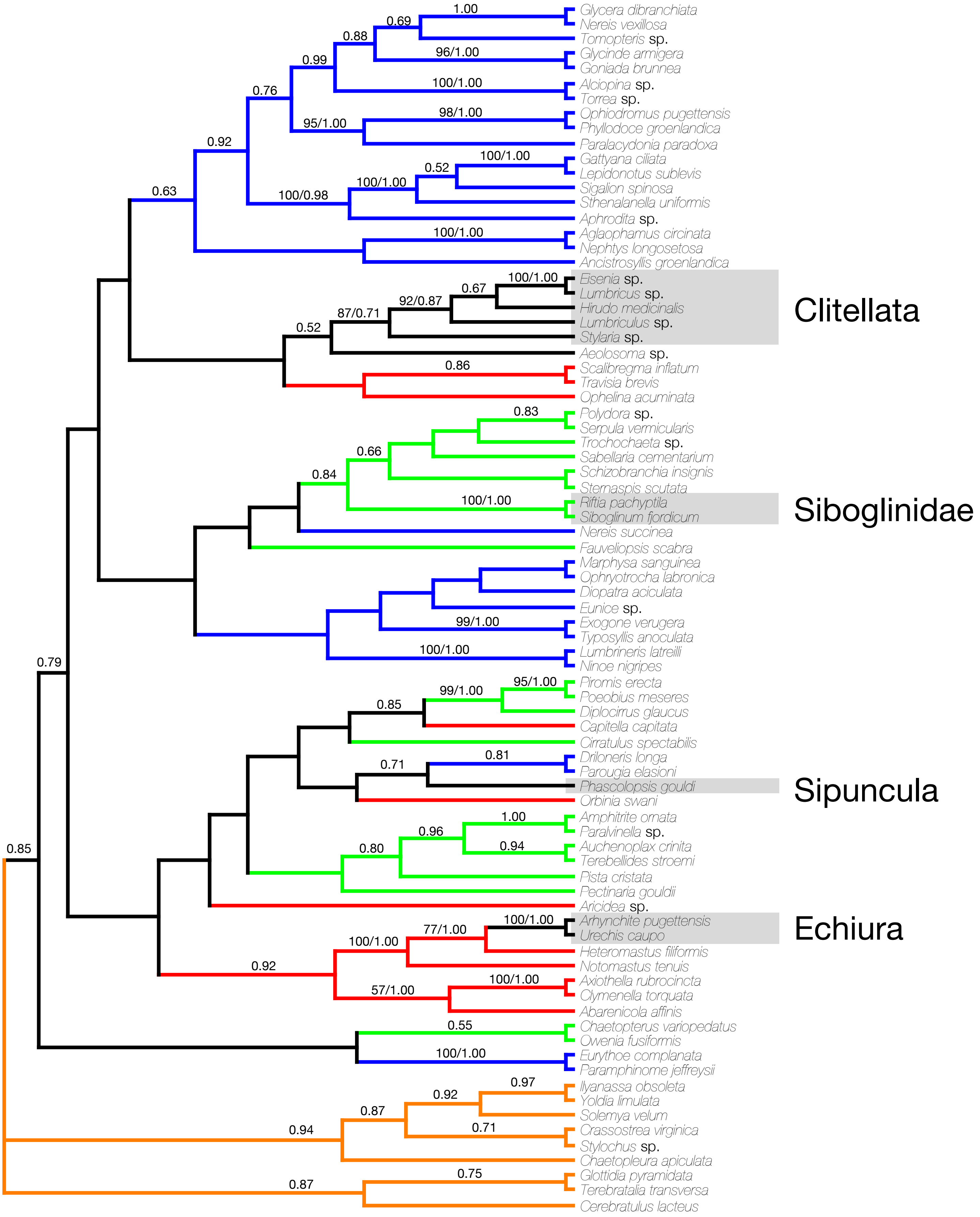

Supplementary Fig. 1. Cladogram of ML analysis and BI of 28S partition with 81 taxa (-ln L = 34,291.15). 28S consisted of 5,832 characters, from which 2,504 unambiguously aligned and non-saturated ones were included. BS values above 50 shown at the branches on the left; PP's on the right or alone. ML settings: Base frequencies: A = 0.2484, C = 0.2505, G = 0.2876, T = 0.2135; Rate matrix: AC, AT, CG, GT = 1.0000, AG = 2.8403, CT = 6.2058; α = 0.452; Proportion of invariant sites = 0.2367. Models in BI: GTR+I+Γ. Clitellata, Echiura, Siboglinidae, Sipuncula highlighted with gray, Aciculata = blue, Canalipalpata = green, Scolecida = red, Outgroup = orange.
