# Supplementary material for: Annelid phylogeny and the status of Sipuncula and Echiura
Source: BMC Evol Biol. 2007 Apr 5;7:57. doi: 10.1186/1471-2148-7-57 (PMC1855331; doi:10.1186/1471-2148-7-57)
Supplement: Additional file 2 — ML tree of 18S rRNA partition. This file contains the result of the phylogenetic reconstruction of the 18S rRNA partition with 81 OTUs. [file 1471-2148-7-57-S2.pdf]

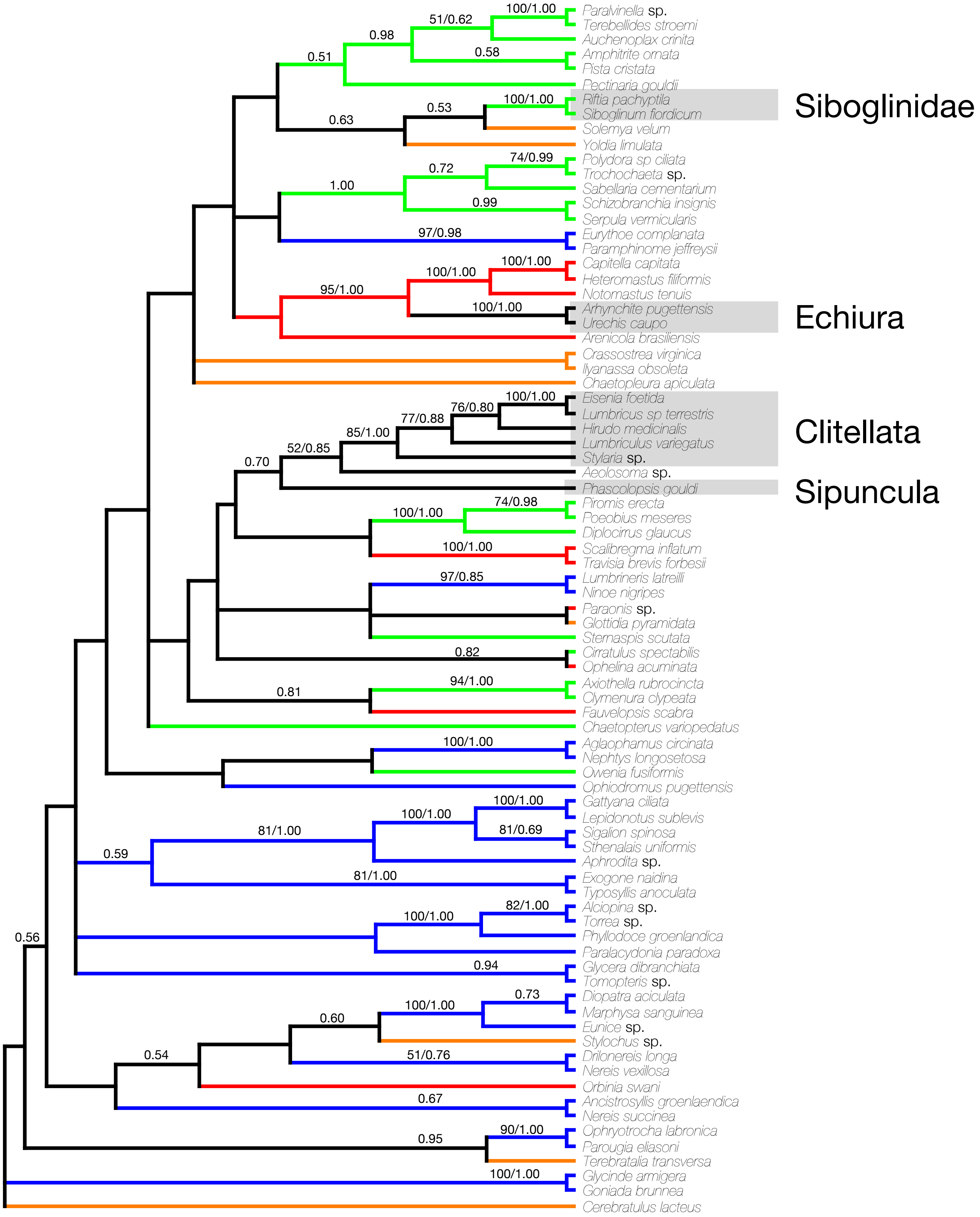

Supplementary Fig. 2. Cladogram of ML analysis and BI of nucSSU partition with 81 OTUs (-ln L = 15,094.94). nucSSU consisted of 2,376 characters, from which 1,375 unambiguously aligned and non-saturated ones were included. BS values above 50 shown at the branches on the left; PP's on the right or alone. ML settings: Base frequencies: A = 0.2536, C = 0.2362, G = 0.2677, T = 0.2425; Rate matrix: AC, AT, CG, GT = 1.0000, AG = 3.4196 CT = 3.9351; α = 0.581; Proportion of invariant sites = 0.3710. Models in BI: GTR+I+Γ. Clitellata, Echiura, Siboglinidae, Sipuncula highlighted with grey, Aciculata = blue, Canalipalpata = green, Scolecida = red, Outgroup = orange.
