# Supplementary material for: Annelid phylogeny and the status of Sipuncula and Echiura
Source: BMC Evol Biol. 2007 Apr 5;7:57. doi: 10.1186/1471-2148-7-57 (PMC1855331; doi:10.1186/1471-2148-7-57)
Supplement: Additional file 3 — ML tree of EF1α partition. This file contains the result of the phylogenetic reconstruction of the EF1α partition with 81 OTUs. [file 1471-2148-7-57-S3.pdf]

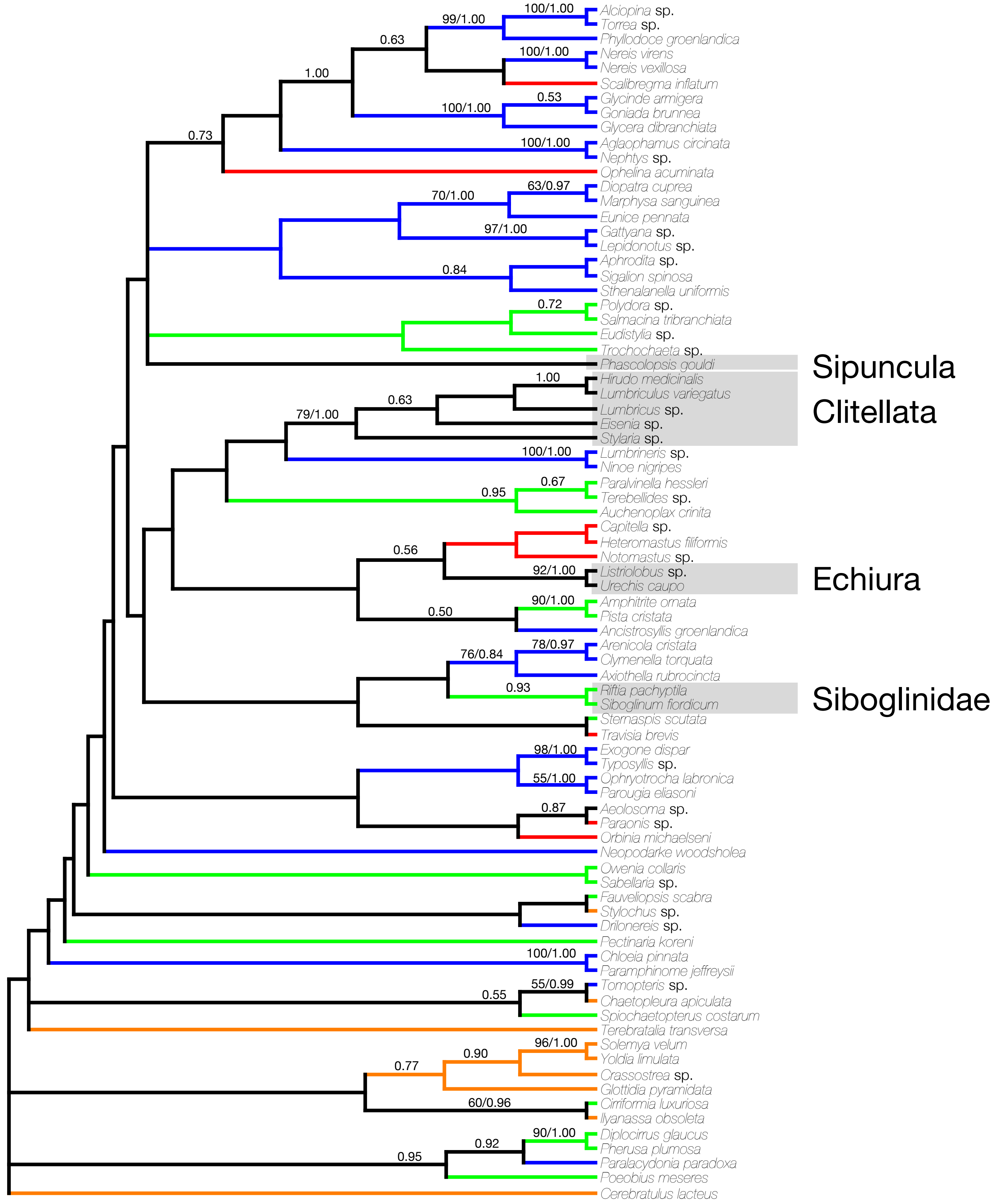

Supplementary Fig. 3. Strict consensus tree of 2 best ML trees and BI of EF1α partition with 81 OTUs (-ln L = 14,231.29). EF1α consisted of 1,274 characters, from which 673 unambiguously aligned and non-saturated ones were included. BS values above 50 shown at the branches on the left; PP's on the right or alone. ML settings: Base frequencies: A = 0.3618, C = 0.2314, G = 0.2263, T = 0.1805; transition/transversion ratio K = 1.368288; α = 0.6642; Proportion of invariant sites = 0.3964. Models in BI: GTR+I+Γ. Clitellata, Echiura, Siboglinidae, Sipuncula highlighted with grey, Aciculata = blue, Canalipalpata = green, Scolecida = red, Outgroup = orange.
