# Supplementary material for: Annelid phylogeny and the status of Sipuncula and Echiura
Source: BMC Evol Biol. 2007 Apr 5;7:57. doi: 10.1186/1471-2148-7-57 (PMC1855331; doi:10.1186/1471-2148-7-57)
Supplement: Additional file 4 — BI tree of the NucMt data set with amino acid partitions. This file contains the result of the phylogenetic reconstruction of the NucMt data set with 10 OTUs and protein-coding genes translated into amino acid sequences using the genetic standard code for EF1α and the mitochondrial invertebrate code for the mitochondrial genes ATP8, COX1-3, CYTB, and NAD6. [file 1471-2148-7-57-S4.pdf]

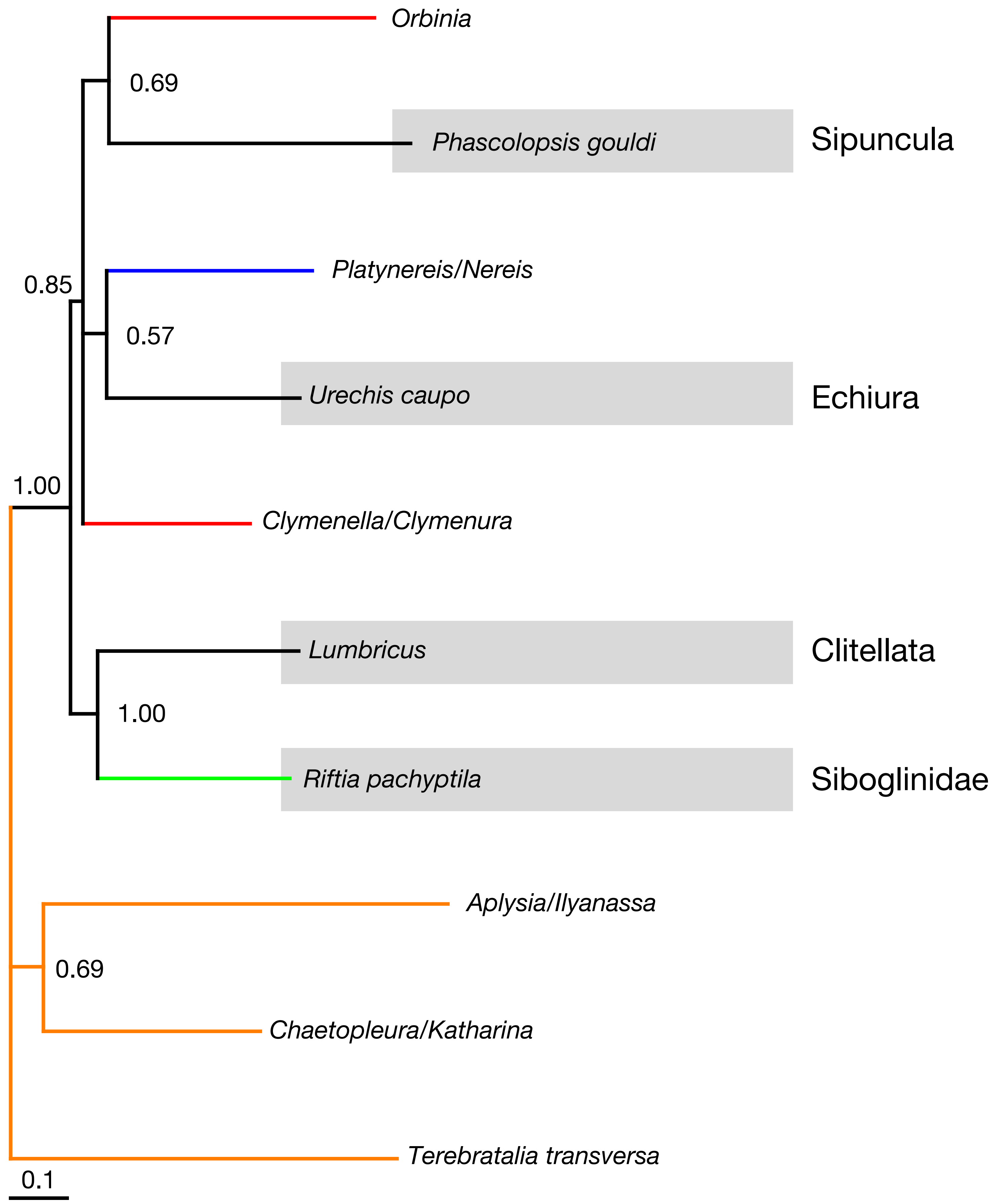

Supplementary Fig. 4. Cladogram of BI of NucMt data set with 10 OTUs and amino acids (-ln L = 39,898.71). NucMt with amino acids consisted of 12,347 characters, from which 6,638 unambiguously aligned and non-saturated ones were included. PP's above 50 shown. Models in BI: nucLSU, mtSSU: GTR+I+Γ; nucSSU: K80+I+Γ; mtLSU = GTR+Γ; Ef1a, ATP8, COX1-3, CYTB, NAD6 = Protein model mixture. Clitellata, Echiura, Siboglinidae, Sipuncula highlighted with gray, Aciculata = blue, Canalipalpata = green, Scolecida = red, Outgroup = orange.
