# Supplementary material for: Annelid phylogeny and the status of Sipuncula and Echiura
Source: BMC Evol Biol. 2007 Apr 5;7:57. doi: 10.1186/1471-2148-7-57 (PMC1855331; doi:10.1186/1471-2148-7-57)
Supplement: Additional file 5 — List of taxa. This file contains a list of taxa used in the phylogenetic analyses. [file 1471-2148-7-57-S5.pdf]

Supplementary Table 1. List of taxa used in the analyses with nucSSU, nucLSU and EF1α sequence as well as the mitochondrial genome accession numbers, determined sequences are in bold. Localities and voucher numbers are attached with the accession number and are only given for taxa from which new sequences were determined.

Taxon	Species	nucSSU	nucLSU	EF1α	mtDNA
Arenicolidae	*Arenicola brasiliensis* (Nonato, 1958)	**DQ790076**			
	Abarenicola affinis (Ashworth, 1903)		**DQ790025**		
	Arenicola cristata Stimpson, 1855			**DQ813351**	
Capitellidae	*Heteromastus filiformis* (Claparede, 1864)	**DQ790081**	**DQ790038**	**DQ813369**	
	Notomastus tenuis Moore, 1909	**DQ790084**	**DQ790044**		
	Notomastus sp.			**DQ813379**	
	Capitella capitata (Fabricius, 1780)	AF508118	**DQ790028**		
	Capitella sp.			AB003706	
Maldanidae	*Clymenella torquata* (Leidy, 1855)		**DQ790030**	**DQ813356**	NC006321
	Clymenura clypeata (Saint-Joseph, 1894)	AF448152			
	Axiothella rubrocincta (Johnson, 1901)	**DQ790078**	**DQ790027**	**DQ813353**	
Opheliidae	*Ophelina acuminata* Oersted, 1843	**DQ790085**	**DQ790045**	AB003708	
Orbiniidae	*Orbinia swani* Pettibone, 1957	**DQ790087**	**DQ790048**		
	Orbinia michaelseni			**DQ813381**	
	Orbinia latreilli (Audouin & Edwards, 1833)				AY961084
Paranoidae	*Paraonis* sp.	**DQ790090**		**DQ813386**	
	Aricidea sp.		**DQ790052**		
Scalibregmatidae	*Scalibregma inflatum* Rathke, 1843	**DQ790093**	**DQ790060**	**DQ813397**	
	Travisia brevis Moore, 1923		**DQ790069**	**DQ813407**	
	Travisia forbesii Johnston, 1840	AF508127			
Aphroditidae	*Aphrodita* sp.	AY894295	**DQ790024**	**DQ813350**	
Polynoidae	*Gattyana ciliata* Moore, 1902	AY894297	**DQ790035**		
	Gattyana sp.			**DQ813365**	
	Lepidonotus sublevis Verrill, 1873	AY894301	**DQ790039**		
	Lepidonotus sp.			**DQ813370**	
Sigalionidae	*Sigalion spinosus* (Hartman, 1939)	AY894304	**DQ790062**	**DQ813399**	
	Sthenelanella uniformis Moore, 1910	AY894306	**DQ790064**	**DQ813402**	
Hesionidae	*Ophiodromus pugettensis* (Johnson, 1901)	**DQ790086**	**DQ790046**		
	Neopodarke woodsholea Hartman, 1965			**DQ813375**	
Nereididae	*Nereis succinea* (Frey and Leuchart, 1847)	AY210447	AY210464		
	Nereis virens (M. Sars, 1835)			U90064	
	Nereis vexillosa Grube, 1851	**DQ790083**	**DQ790043**	**DQ813377**	
	Platynereis dumerlii				NC000931
Pilargidae	*Ancistrosyllis groenlandica* McIntosh, 1879	**DQ790075**	**DQ790023**	**DQ813349**	
Syllidae	*Exogone naidina* Oersted, 1845	AF474290			
	Exogone verugera (Claparede, 1868)		**DQ790033**		
	Exogone dispar (Webster, 1879)			**DQ813363**	
	Typosyllis anoculata (Hartmann-Schröder, 1962)	**DQ790098**	**DQ790071**		
	Typosyllis sp.			**DQ813409**	
Glyceridae	*Glycera dibranchiata* Ehlers, 1868	AY995208	AY995207	**DQ813366**	
Goniadidae	*Goniada brunnea* Treadwell, 1906	**DQ790080**	**DQ790037**	**DQ813368**	
	Glycinde armigera Moore, 1911	**DQ790079**	**DQ790036**	**DQ813367**	
Nephtyidae	*Nephtys longosetosa* (Oersted, 1842)	**DQ790082**	**DQ790042**		
	Nephtys sp.			**DQ813376**	
	Aglaophamus circinata (Verrill, 1874)	**DQ790072**	**DQ790020**	**DQ813346**	

Taxon	Species	nucSSU	nucLSU	EF1α	mtDNA
Paralacydoniidae	*Paralacydonia paradoxa* Fauvel, 1913	**DQ790088**	**DQ790050**	**DQ813384**	
Phyllodocidae	*Phyllodoce groenlandica* (Oersted, 1843)	**DQ790092**	**DQ790055**	**DQ813389**	
Alciopidae	*Alciopina* sp.	**DQ790073**	**DQ790021**	**DQ813347**	
	Torrea sp.	**DQ790096**	**DQ790068**	**DQ813406**	
Tomopteridae	*Tomopteris* sp.	**DQ790095**	**DQ790067**	**DQ813405**	
Amphinomidae	*Paramphinome jeffreysii* (Mcintosh, 1868)	AY838856	AY838865	**DQ813383**	
	Eurythoë complanata (Pallas, 1766)<br>*Chloeia pinnata* Moore, 1911	AY364851	AY364849	 **DQ813354**	
Dorvilleidae	*Ophryotrocha labronica* La Greca & Bacci, 1962	AY838855	**DQ790047**	**DQ813380**	
	Parougia eliasoni (Oug, 1978)	AF412798	**DQ790053**	**DQ813387**	
Eunicidae	*Marphysa sanguinea* (Montagu, 1815)	AY525621	AY838861	**DQ813374**	
	Eunice sp.<br>*Eunice pennata* (O.F. Müller, 1776)	AF412791	AY732229	 **DQ813362**	
Lumbrineridae	*Lumbrineris latreilli* Audouin & Milne-Edwards, 1834<br>*Lumbrineris* sp.	AY525623	AY366512	 **DQ813373**	
	Ninoe nigripes Pettibone, 1982	AY838852	AY838862	**DQ813378**	
Oenonidae	*Driloneris longa* Webster, 1879<br>*Driloneris* sp.	AY838847	AY838860	 **DQ813359**	
Onuphidae	*Diopatra aciculata* Knox and Cameron, 1971<br>*Diopatra cuprea* (Bosc, 1802)	AY838845	AY838858	 **DQ813357**	
Oweniidae	*Owenia fusiformis* delle Chiaje, 1841<br>*Owenia collaris* Hartman, 1955	AF448160	**DQ790049**	 **DQ813382**	
Sabellaridae	*Sabellaria cementarium* Moore, 1906<br>*Sabellaria* sp.	AY732223	AY732226	 **DQ813395**	
Sabellidae	*Schizobranchia insignis* Bush, 1905<br>*Eudistylia* sp.	AY732222	AY732225	 **DQ813361**	
Serpulidae	*Serpula vermicularis* Linnaeus, 1767<br>*Salmacina tribranchiata* (Moore, 1923)	AY732224	AY732227	 **DQ813396**	
Siboglinidae	*Riftia pachyptila* Jones, 1981	AF168739	Z21534	**DQ813394**	AY741642
	Siboglinum fiordicum Webb, 1963	X79876	**DQ790061**	**DQ813398**	
Cirratulidae	*Cirratulus spectabilis* (Kinberg, 1866)<br>*Cirriformia luxuriosa* (Moore, 1904)	AY708536	**DQ790029**	 **DQ813355**	
Fauveliopsidae	*Fauveliopsis scabra* Hartman & Fauchald, 1971	AY708537	**DQ790034**	**DQ813364**	
Flabelligeridae	*Diplocirrus glaucus* (Malmgren, 1867)	AY708534	**DQ790031**	**DQ813358**	
	Pherusa plumosa (Müller, 1776)<br>*Piromis erecta*	AY708528	**DQ790056**	 **DQ813390**	
Poeobiidae	*Poeobius meseres* Heath, 1930	AY708526	**DQ790058**	**DQ813392**	
Sternaspidae	*Sternaspis scutata* (Ranzani, 1817)	AY532329	**DQ790063**	**DQ813401**	
Alvinellidae	*Paralvinella* sp.<br>*Paralvinella hessleri* Desbruyères & Laubier, 1989	**DQ790089**	**DQ790051**	 **DQ813385**	
Ampharetidae	*Auchenoplax crinita* Ehlers, 1887	**DQ790077**	**DQ790026**	**DQ813352**	
Pectinariidae	*Pectinaria gouldi* (Verrill, 1873)<br>*Pectinaria koreni* (Malmgren, 1866)	**DQ790091**	**DQ790054**	 **DQ813388**	
Terebellidae	*Amphitrite ornata* (Leidy, 1855)	**DQ790074**	**DQ790022**	**DQ813348**	
	Pista cristata (O. F. Mueller, 1776)	AY611461	**DQ790057**	**DQ813391**	
Trichobranchidae	*Terebellides stroemi* Sars, 1835<br>*Terebellides* sp.	**DQ790094**	**DQ790066**	 **DQ813404**	
Chaetopteridae	*Chaetopterus variopedatus* (Renier, 1804)<br>*Spiochaetopterus costarum* (Claparède, 1870)	U67324	AY145399	 **DQ813400**	
Spionidae	*Polydora ciliata* (Johnston, 1838)<br>*Polydora* sp.	U50971	 **DQ790059**	 **DQ813393**	
Trochochaetidae	*Trochochaeta* sp.	**DQ790097**	**DQ790070**	**DQ813408**	
Clitellata	*Lumbricus terrestris* Linnaeus, 1758<br>*Lumbricus* sp.	AJ272183	 **DQ790041**	 **DQ813372**	NC001673
	Lumbriculus variegatus (Mueller, 1774)<br>*Lumbriculus* sp.	AY040693	 **DQ790040**	AF063422	
	Eisenia foetida Savigny, 1826<br>*Eisenia* sp.	AB076887	 **DQ790032**	**DQ813360**	
	Stylaria sp.	U95946	**DQ790065**	**DQ813403**	

**Taxon**	**Species**	**nucSSU**	**nucLSU**	**EF1α**	**mtDNA**
Clitellata	*Hirudo medicinalis* Linnaeus, 1758	Z83752	AY364866	U90063	
Aeolosomatidae	*Aeolosoma* sp.	Z83748	**DQ790019**	**DQ813345**	
**Possible Annelidan Subtaxa**					
Echiura	*Arhynchite pugettensis* Fisher, 1949	AY210441	AY210455		
	Listriolobus sp.			**DQ813371**	
	Urechis caupo Fisher & MacGinitie 1928	AF119076	AF519268	**DQ813410**	NC006379
Sipuncula	*Phascolopsis gouldi* (Pourtalès, 1851)	AF342796	AF342795	AF063421	AF374337
**Outgroups**					
Bivalvia	*Crassostrea virginica* (Gmelin, 1791)	AB064942	AY145400		
	Crassostrea sp.			**DQ813412**	
	Solemya velum Say, 1822	AF120524	AY145421	**DQ813415**	
	Yoldia limaluta (Say, 1831)	AF120528	AY145424	**DQ813417**	
Gastropoda	*Ilyanassa obsoleta* (Say, 1822)	AY145379	AY145411		
	Ilyanassa sp.			**DQ813414**	
	Aplysia californica J.G. Cooper, 1863				NC005827
Polyplacophora	*Chaetopleura apiculata* (Say, 1834)	AY145370	AY145398	U90062	
	Katharina tunicata (Wood, 1815)				NC001636
Brachiopoda	*Terebratalia transversa* (Sowerby, 1846)	AF025945	AF342802	**DQ813416**	NC000941
	Glottidia pyramidata (Stimpson, 1860)	U12647	AY210459	**DQ813413**	
Nemertea	*Cerebratulus lacteus* (Leidy, 1851)	AY145368	AY145396	**DQ813411**	
Platyhelminthes	*Stylochus* sp.	AF342801	AF342800	AY580253	
