# Supplementary material for: Annelid phylogeny and the status of Sipuncula and Echiura
Source: BMC Evol Biol. 2007 Apr 5;7:57. doi: 10.1186/1471-2148-7-57 (PMC1855331; doi:10.1186/1471-2148-7-57)
Supplement: Additional file 6 — Detection of saturated positions. This file contains further information on amplification of EF1α, alignment procedures, homogeneity of base frequencies and phylogenetic results of data partitions. [file 1471-2148-7-57-S6.pdf]

Supplementary Data

Amplification of EF1α

For EF1α, total RNA was isolated using RNAwiz™ (Ambion) and reverse transcribed using SuperScript™ II (Invitrogen). Amplification of EF1α was carried out using nested degenerate primers [40.6F, 41.2RC, 40.71F designed by Regier and Shultz[1]; and JH16R: 5'-KNRAANKNYTCNACRCACA-3'] with the external and internal pairs being 40.6F /41.2RC and 40.71F/JH16R, respectively. An initial touchdown PCR was performed (from 60°C to 55°C decreasing 1°C per 3 cycles, then from 55°C to 45°C decreasing 0.5°C per cycle for 21 cycles followed by 20 cycles at 45°C), and the products were reamplified at 50°C for 25 cycles.

Alignment and assessing substitutional saturation

Procedures to preliminary analyze our alignments prior to any phylogenetic analysis were slightly modified from the ones described and discussed by Jördens *et al*[2]. To examine the degree of variation more precisely for the structural genes, nuclear and mitochondrial versions of SSU and LSU genes, a sliding window analysis was performed. The percentage of variation within a window of nine bases was determined. The genetic variation is expressed as a percentage of the 27 possible base substitutions in a window of 9 bases. Effectively, this procedure assumes that each substitution is phylogenetically informative (Hennig's auxiliary principle), because the minimally possible number of substitutions to explain the character pattern in the window expresses the genetic variation. For example, if a position exhibits all four nucleotides three substitutions are at least necessary to explain the pattern (e.g., A→C, A→T, A→G). The window is then moved on one position and the percentage of variation is determined anew. For each position the mean is calculated out of the nine windows, which comprise this particular position. Classes of genetic variation were defined in 10% increments ranging from 0-100% (Suppl. Table 3). To obtain more or less the same number of positions per class of variation within the analysis the 10% increments were merged as shown in Suppl. Table 3. This procedure also reflects to some degree a discrete gamma distribution. In contrast to their nuclear counterparts, the mitochondrial SSU and LSU exhibit more variation, and thus they have greater proportional representation in higher classes of variation. Furthermore, the 1st, 2nd and 3rd positions of the protein-coding genes

(EF1α, ATP8, COX 1-3, CYTB, NAD6) were treated as different classes of genetic variation.

Supplementary Table 3: *Classes of genetic variation.* Number of positions in the 10% increments of the sliding window analyses of nucSSU, nucLSU, mtSSU and mtLSU and in the merged increments. Ppi = Positions per increment; Ppmi = Positions per merged increments.

<table>
<tr><th rowspan="2">Increments</th><th colspan="2">nucSSU</th><th colspan="2">nucLSU</th><th colspan="2">mtSSU</th><th colspan="2">mtLSU</th></tr>
<tr><th>Ppi</th><th>Ppmi</th><th>Ppi</th><th>Ppmi</th><th>Ppi</th><th>Ppmi</th><th>Ppi</th><th>Ppmi</th></tr>
<tr><td>0-10%</td><td>328</td><td>328</td><td>351</td><td>351</td><td>17</td><td rowspan="3">184</td><td>34</td><td rowspan="3">243</td></tr>
<tr><td>10-20%</td><td>323</td><td>323</td><td>431</td><td>431</td><td>60</td><td>86</td></tr>
<tr><td>20-30%</td><td>199</td><td>199</td><td>453</td><td>453</td><td>107</td><td>123</td></tr>
<tr><td>30-40%</td><td>207</td><td>207</td><td>419</td><td>419</td><td>82</td><td rowspan="3">222</td><td>116</td><td rowspan="2">220</td></tr>
<tr><td>40-50%</td><td>206</td><td>206</td><td>305</td><td>305</td><td>68</td><td>104</td></tr>
<tr><td>50-60%</td><td>112</td><td>112</td><td>313</td><td>313</td><td>72</td><td>69</td><td rowspan="5">86</td></tr>
<tr><td>60-70%</td><td>99</td><td rowspan="4">144</td><td>232</td><td>232</td><td>65</td><td rowspan="4">75</td><td>14</td></tr>
<tr><td>70-80%</td><td>31</td><td>106</td><td rowspan="3">153</td><td>5</td><td>3</td></tr>
<tr><td>80-90%</td><td>14</td><td>34</td><td>5</td><td>0</td></tr>
<tr><td>90-100%</td><td>0</td><td>13</td><td>0</td><td>0</td></tr>
</table>

Homogeneity of base frequencies, uncorrected substitution rates as well as transition (= TI) to transversion (TV) ratio were estimated for each of the classes of variation to determine saturated positions. Using the options “pairdiff”, “include”, and “exclude” of PAUP4.0b^{3}, a pair wise comparison of the aligned sequences is performed for each class of variation. To detect saturation the TI/TV ratios have been plotted against the uncorrected distances p. Due to both graphical convenience and better comparability between the different genes the plot only displays the mean values of the TI/TV ratios and genetic distances p as well as their corresponding standard deviation (Suppl. Fig. 5). Suppl. Table 4 summarizes the homogeneity of base frequencies for the different genes and their classes of genetic variation.

Overall the results for the structural genes nucSSU, nucLSU, mtSSU and mtLSU are similar independent of the genomic origin. With increasing genetic variation the TI/TV ratios converge on an equilibrium indicating saturation of transitions. The standard deviation for TI/TV ratios decreases whereas the standard deviation of genetic distance increases (Suppl. Fig. 5). Although homogeneity of base frequencies is given for nearly all classes of variations in the different genes (except 60-100% of mtSSU; Suppl. Table 4) we excluded the highest

class of genetic variation (50-100%, 60-100%, 70-100%, respectively) in each of the four genes due to the indication of saturation (Suppl. Fig. 5).

In the case of the nuclear protein-coding gene EF1α saturation and heterogeneity of base frequencies can be detected in the 3rd positions and thus they were excluded.

For the 1st positions of the mitochondrial protein-coding genes (ATP8, COX 1-3, CYTB, NAD6) convergence of TI/TV ratios can be shown and thus an indication of saturation of transitions. Furthermore, their 3rd positions exhibit a cloud-like relationship of TI/TV ratios and genetic distance p. In combination with extremely high genetic distances p of about 0.6 this is indicative for additionally saturated transversions. Furthermore, homogeneity of base frequencies is not given for the 1st and 3rd positions of these genes. Thus, we excluded both 1st and 3rd positions of ATP8, COX 1-3, CYTB and NAD6.

Supplementary Table 4: *Homogeneity of base frequencies.* P values of the classes of genetic variation of the different genes used. Significant differences ($p < 0.05$) in bold.

<table>
<tr><th>Increments</th><th>nucSSU</th><th>nucLSU</th><th>mtSSU</th><th>mtLSU</th></tr>
<tr><td>0-10%</td><td>1.00</td><td>1.00</td><td rowspan="3">0.99</td><td rowspan="3">0.99</td></tr>
<tr><td>10-20%</td><td>1.00</td><td>1.00</td></tr>
<tr><td>20-30%</td><td>1.00</td><td>1.00</td></tr>
<tr><td>30-40%</td><td>1.00</td><td>1.00</td><td rowspan="3">0.30</td><td rowspan="2">0.21</td></tr>
<tr><td>40-50%</td><td>1.00</td><td>1.00</td></tr>
<tr><td>50-60%</td><td>1.00</td><td>1.00</td><td rowspan="5">0.10</td></tr>
<tr><td>60-70%</td><td rowspan="4">1.00</td><td>1.00</td><td rowspan="4"><0.01</td></tr>
<tr><td>70-80%</td><td rowspan="3">0.99</td></tr>
<tr><td>80-90%</td></tr>
<tr><td>90-100%</td></tr>
</table>

Position	EF1α	ATP8	COX1	COX2
1st	1.00	**<0.01**	**<0.01**	**<0.01**
2nd	1.00	0.98	0.99	0.99
3rd	**<0.01**	**<0.01**	**<0.01**	**<0.01**

Position	COX3	CYTB	NAD6
1st	**0.02**	**0.03**	**<0.01**
2nd	0.99	0.99	**0.03**
3rd	**<0.01**	**<0.01**	**<0.01**

Phylogenetic results of partitions

Topologies produced by ML and BI are similar or identical within data sets; however, the best trees differed among the individual partitions and combined data sets. Individual partitions are congruent with previous studies based on single genes in that they are inconclusive for higher-level relationships concerning Annelida, Clitellata, and Sipuncula[4,5]. Therefore, we focus mainly on results of combined data sets. Even though trees obtained by the two data sets (Nuc and NucMt) are not congruent with each other, most conclusions regarding the affiliation of major groups like Sipuncula and Clitellata to polychaetes are the same, especially considering explicit hypotheses tested. Due to taxon sampling, the NucMt

data set is limited concerning inference of intra-polychaete relationships and conclusions herein are based on the more inclusive Nuc data set with 45 polychaete "families".

References

1. Regier, J. & Shultz, J. Molecular phylogeny of the major arthropod groups indicates polyphyly of crustaceans and a new hypothesis for the origin of hexapods. *Mol. Biol. Evol.* **14**, 902-913 (1997).
2. Jördens, J., Struck, T. H. & Purschke, G. Phylogenetic inference of Parergodrilidae and *Hrabeiella periglandulata* ("Polychaeta", Annelida) based on sequences of the CO I, 18S- and 28S-rDNA. *J. Zool. Syst. Evol. Res.* **42**, 270-280 (2004).
3. Swofford, D. L. (Sinauer Associates, Sunderland, MA, 2002).
4. Struck, T. H. & Purschke, G. The sistergroup relationship of Aeolosomatidae and Potamodrilidae - a molecular phylogenetic approach based on 18S rDNA and Cytochrome Oxidase I. *Zool. Anz.* **243**, 281-293 (2005).
5. Hall, K. A., Hutchings, P. A. & Colgan, D. J. Further phylogenetic studies of the Polychaeta using 18S rDNA sequence data. *J. Mar. Biol. Ass. UK* **84**, 949-960 (2004).

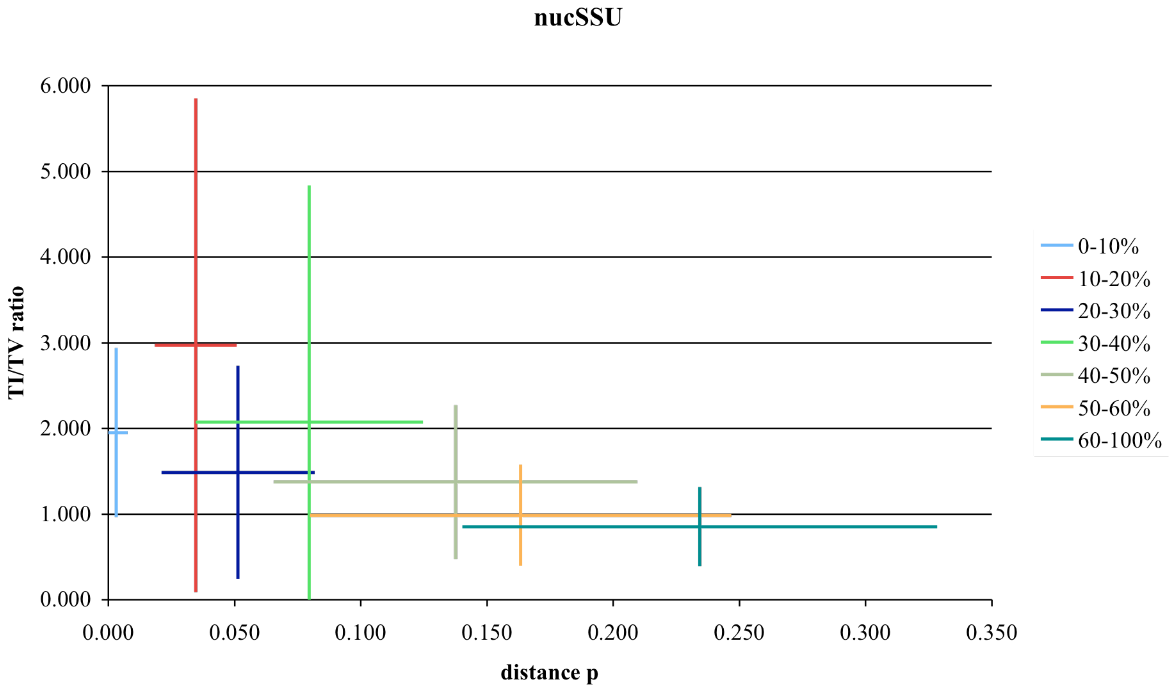

Supplementary Fig. 5. Plots of mean values of the TI/TV ratios and genetic p distances p as well as their corresponding standard deviation.

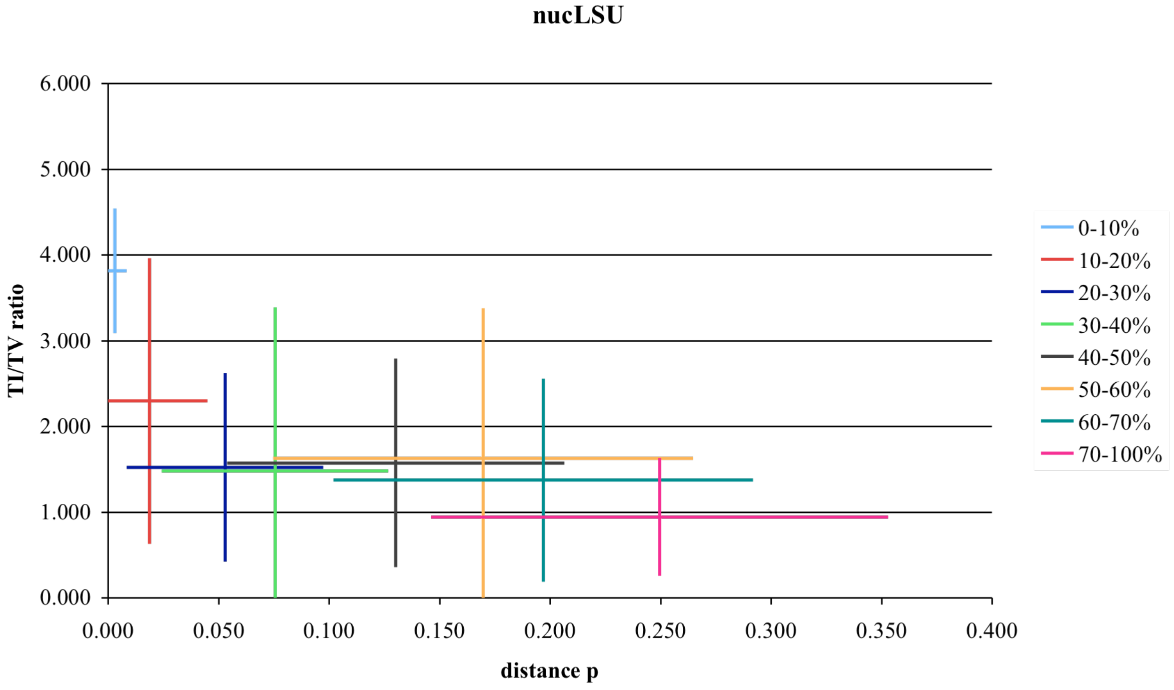

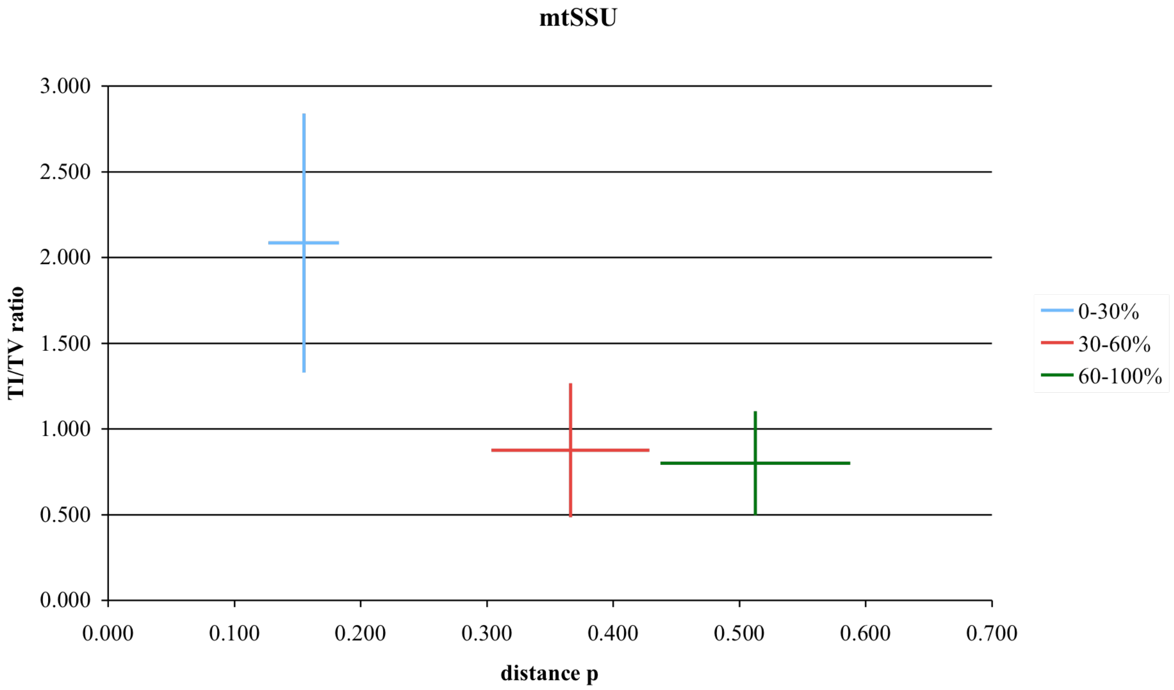

Suppl. Fig. 5. Continued.

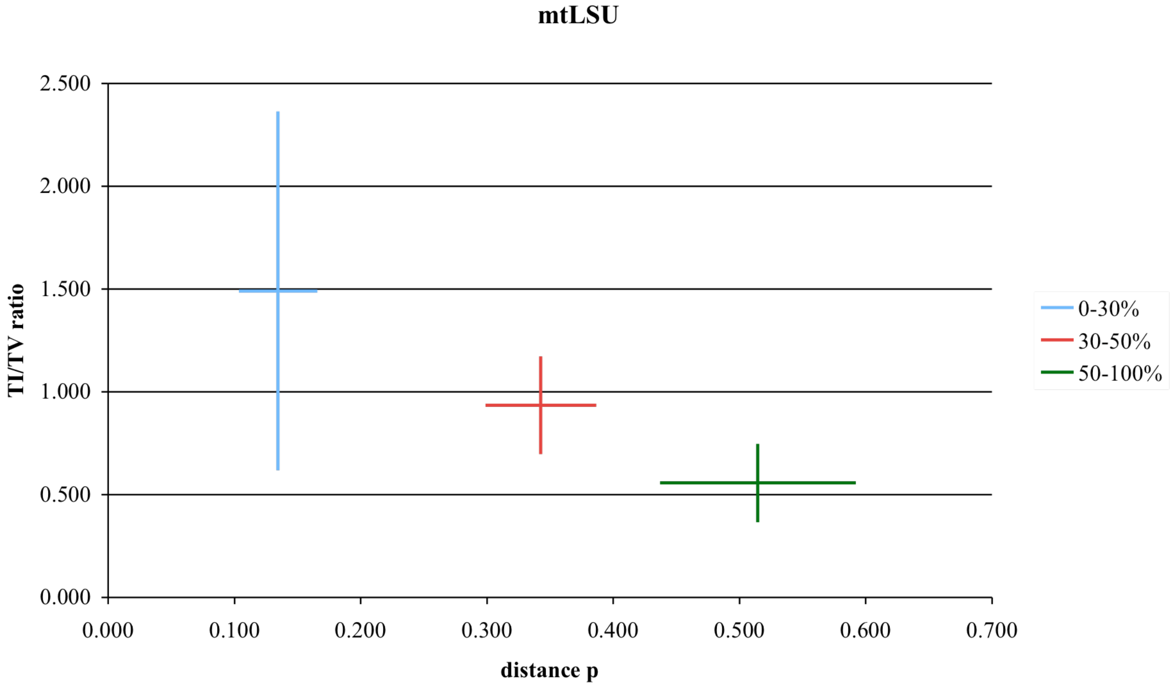

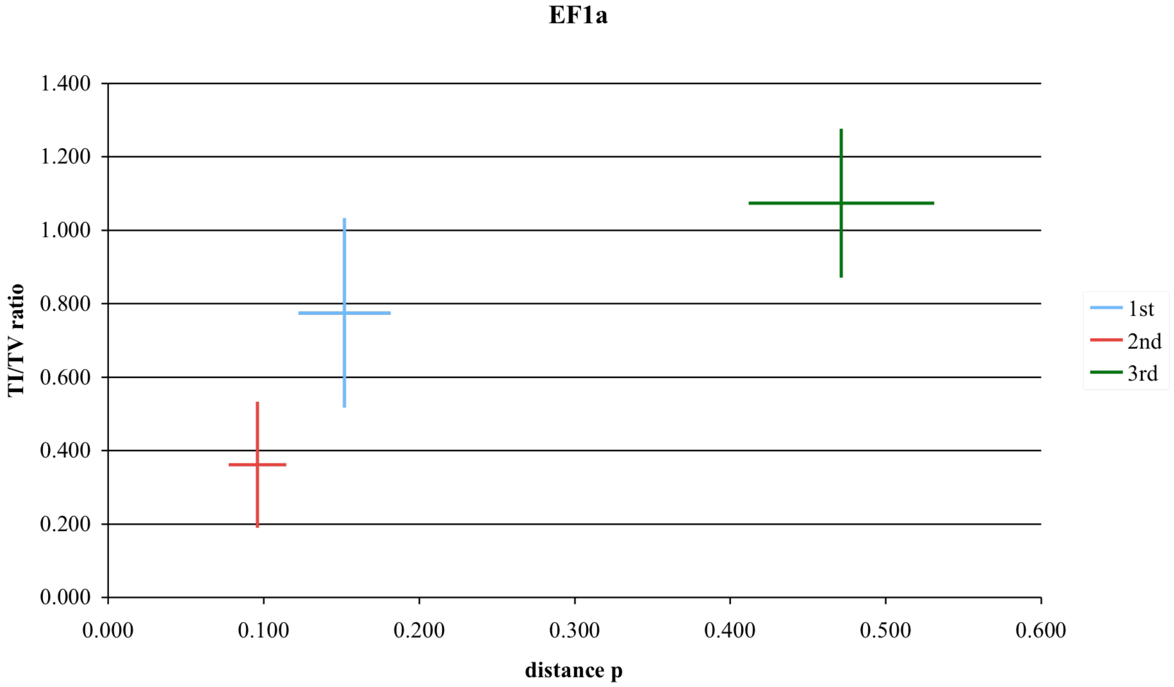

Suppl. Fig. 5. Continued.

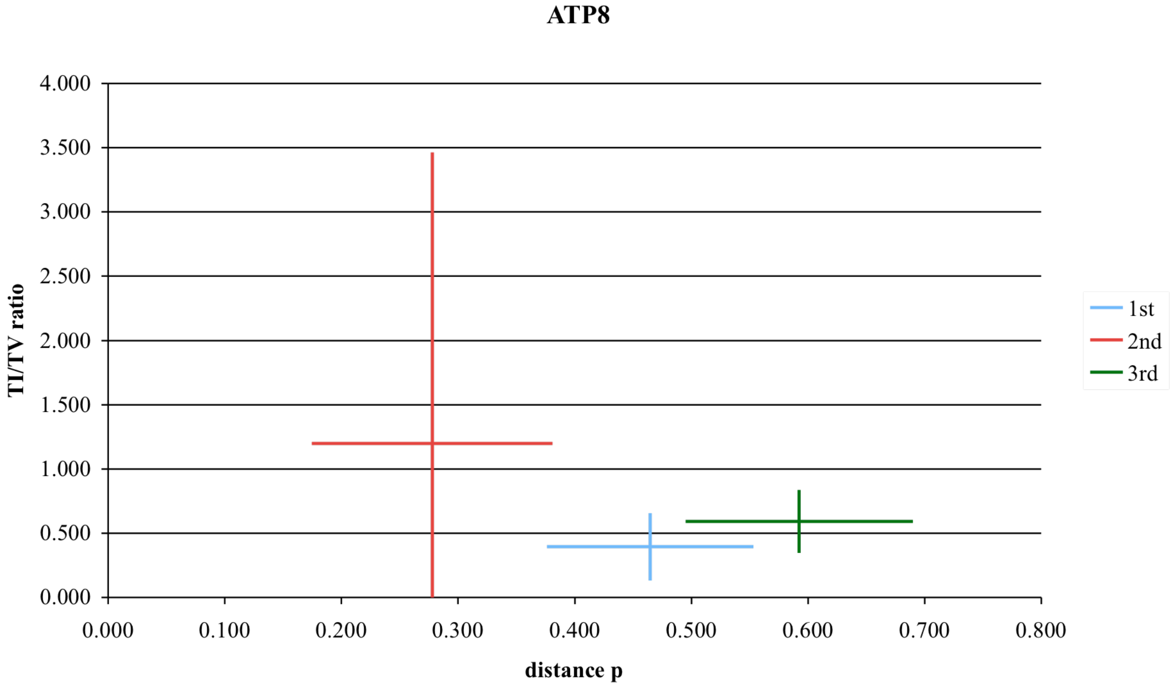

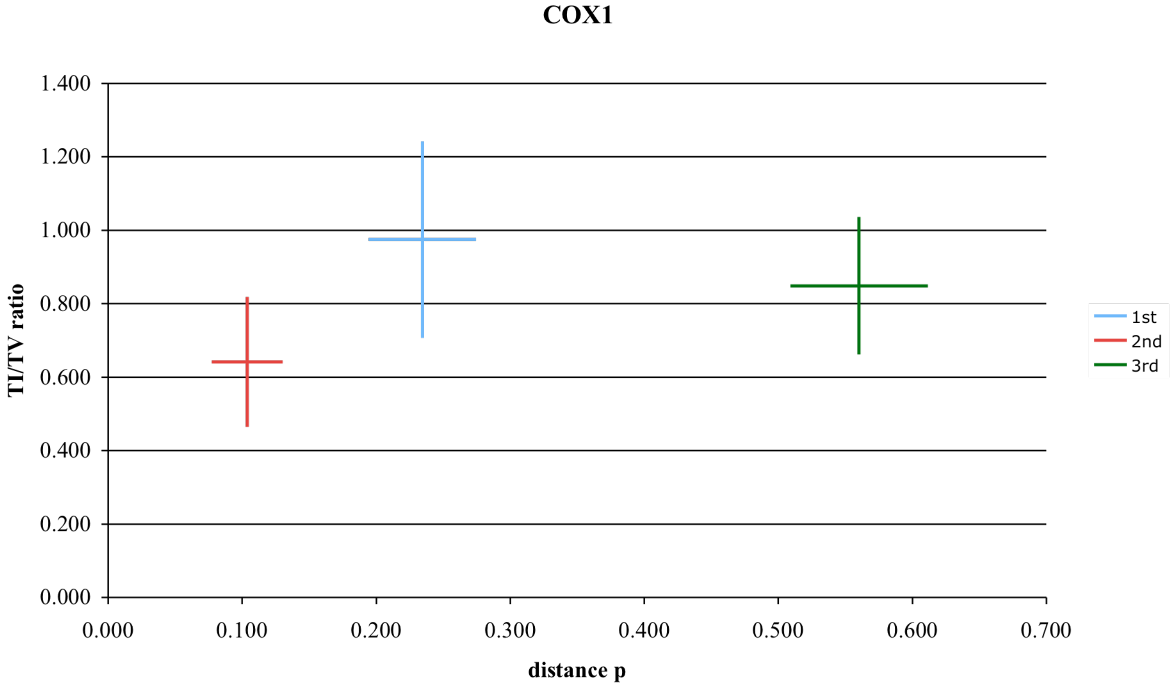

Suppl. Fig. 5. Continued.

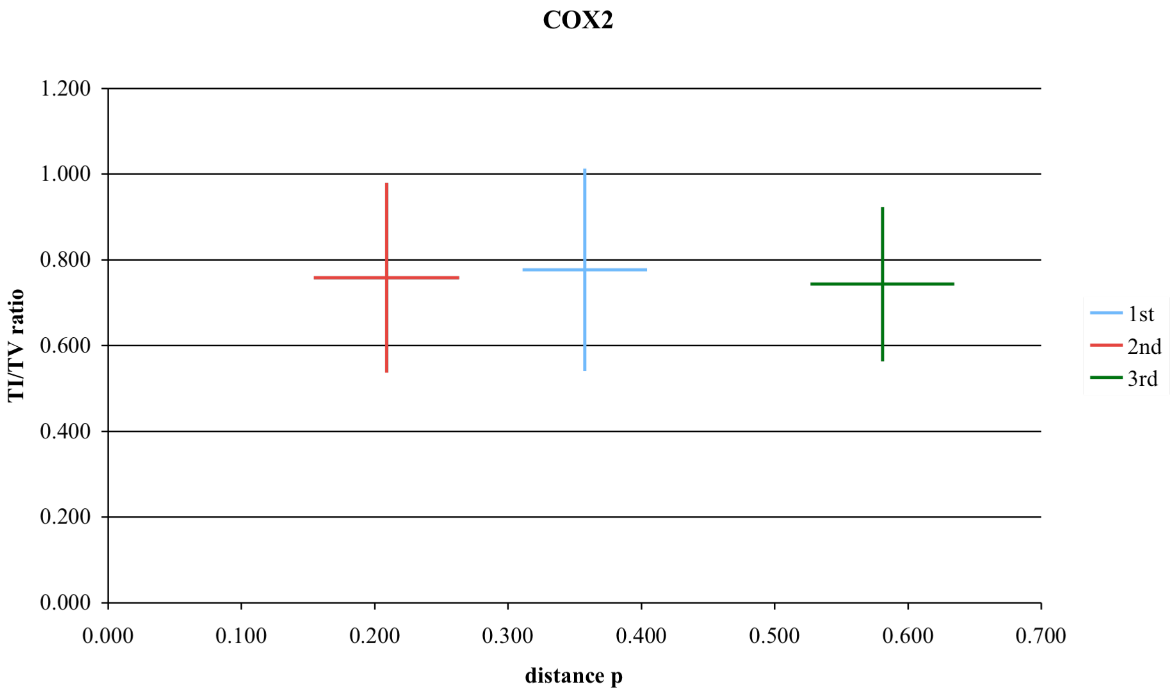

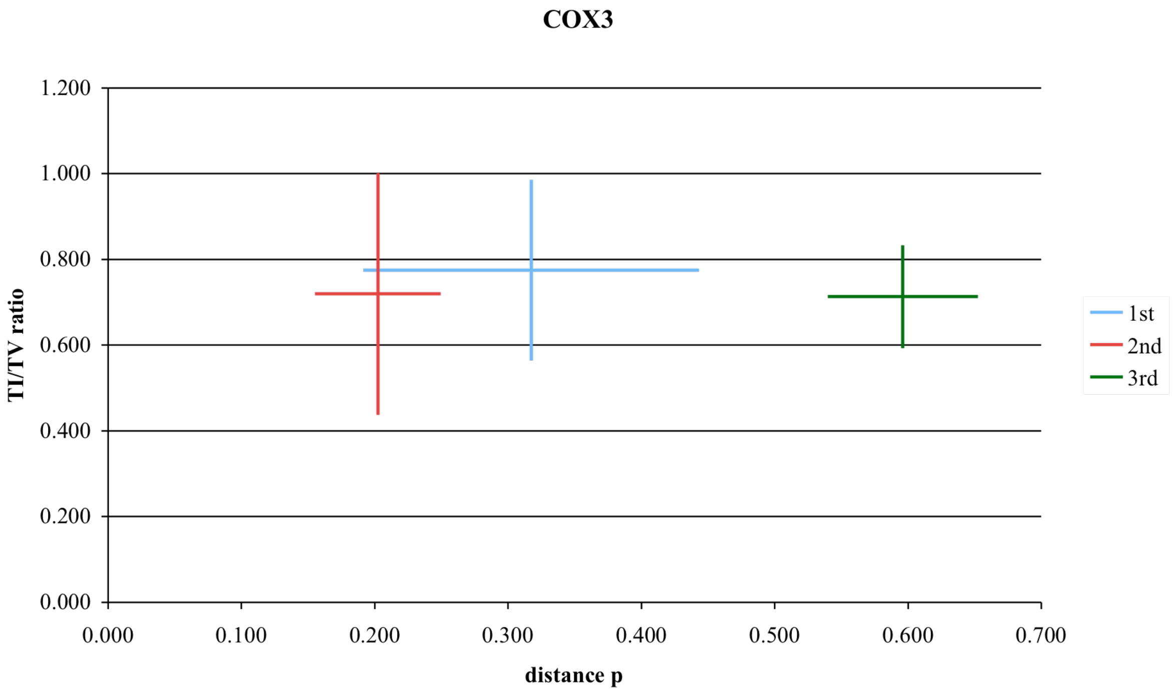

Suppl. Fig. 5. Continued.

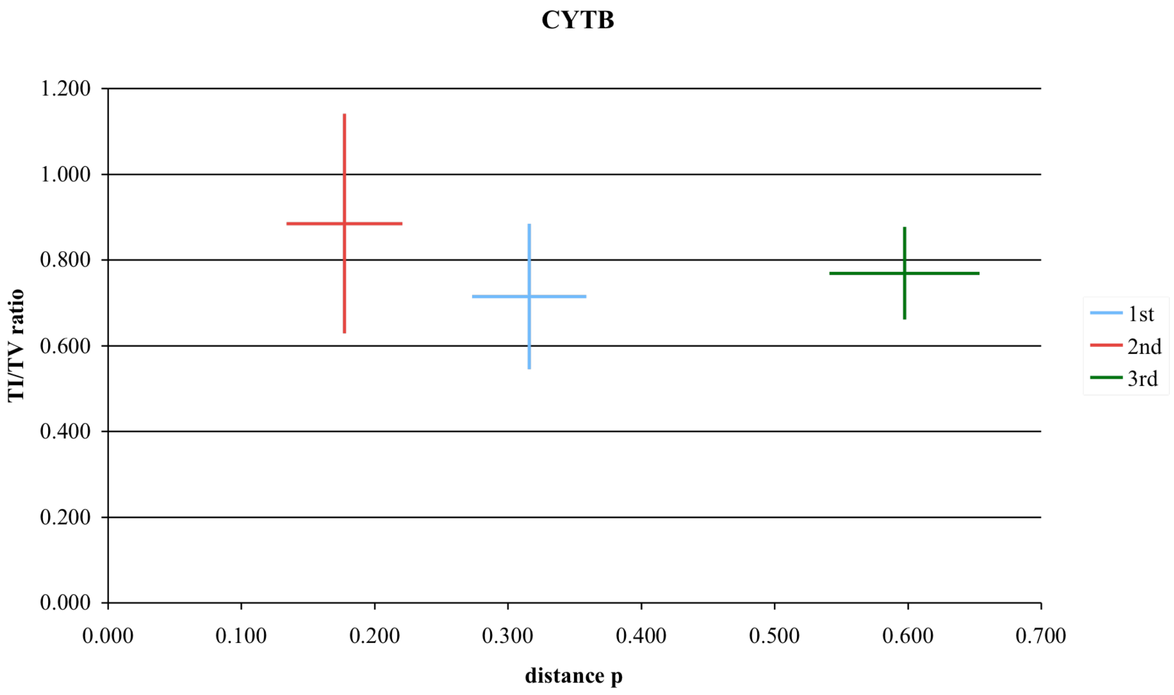

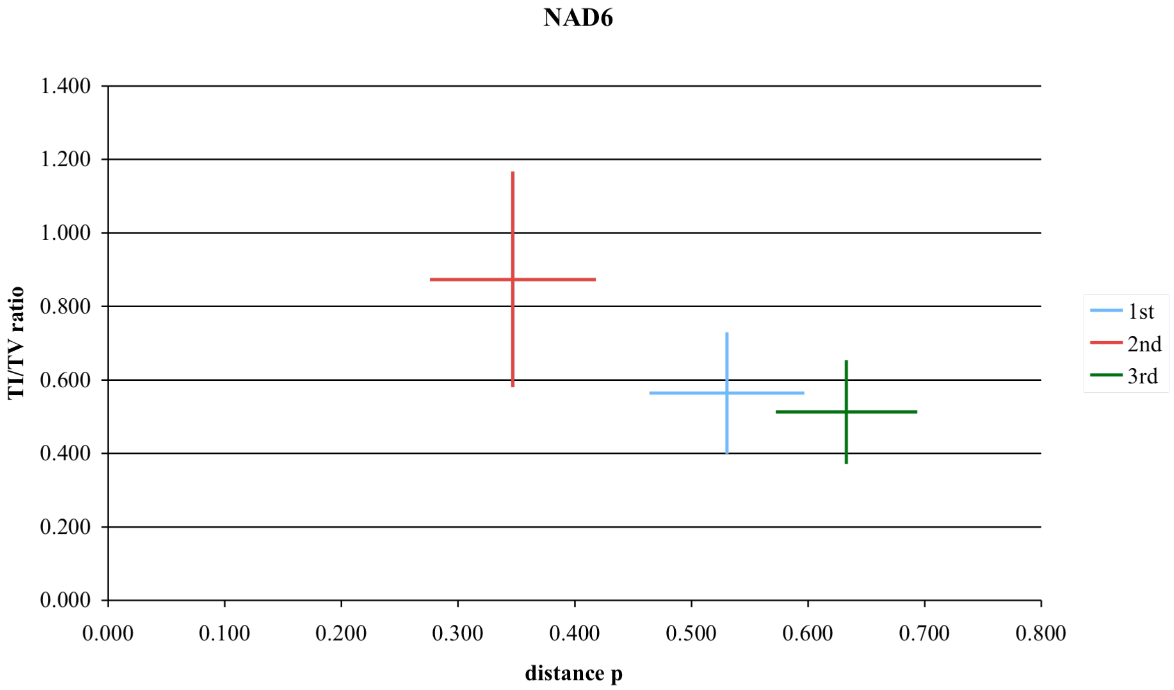

Suppl. Fig. 5. Continued.
