# Supplementary material for: Annelid phylogeny and the status of Sipuncula and Echiura
Source: BMC Evol Biol. 2007 Apr 5;7:57. doi: 10.1186/1471-2148-7-57 (PMC1855331; doi:10.1186/1471-2148-7-57)
Supplement: Additional file 7 — Substitution models used in analyses. This file contains a list of Models used in Maximum Likelihood and Bayesian Inference analyses. [file 1471-2148-7-57-S7.pdf]

Supplementary Table 2. Models used in Maximum Likelihood (ML) and Bayesian Inference (BI) analyses of each partition and data set and the corresponding settings.

Partition/ data set	Model of BI	Model of ML	ML settings												
			Base frequencies				Substitution rates						Ti/Tv ratio K	Shape parameter α	Proportion of invariant sites
			A	C	G	T	A↔C	A↔G	A↔T	C↔G	C↔T	G↔T			
nucLSU	GTR+I+Γ	TrN+I+Γ	0.2484	0.2505	0.2876	0.2135	1.0000	2.8403	1.0000	1.0000	6.2058	1.0000		0.4520	0.2367
nucSSU	GTR+I+Γ	TrN+I+Γ	0.2536	0.2362	0.2677	0.2425	1.0000	3.4196	1.0000	1.0000	3.9351	1.0000		0.5810	0.3710
EF1α	GTR+I+Γ	HKY+I+Γ	0.3618	0.2314	0.2263	0.1805							0.6984	0.6642	0.3964
Nuc		TrN+I+Γ	0.2727	0.2495	0.2586	0.2192	1.0000	2.5097	1.0000	1.0000	3.7263	1.0000		0.4830	0.3103
NucMt		GTR+I+Γ	0.2536	0.2243	0.2498	0.2723	2.3662	4.2648	2.0966	2.5643	6.7166	1.0000		0.4824	0.3555
mtSSU	GTR+I+Γ														
mtLSU	GTR+Γ														
ATP8	F81+Γ														
COX1	F81+Γ														
COX2	F81+Γ														
COX3	F81+Γ														
CYTB	GTR+Γ														
NAD6	F81+Γ														
nucSSU (10 OTU's)	K80+I+Γ														
nucLSU (10 OTU's)	GTR+I+Γ														
EF1α (10 OTU's)	F81+I+Γ														
